# Photocatalytic Hydrogen Generation by Vesicle‐Embedded [FeFe]Hydrogenase Mimics: A Mechanistic Study

**DOI:** 10.1002/chem.201902514

**Published:** 2019-09-26

**Authors:** René Becker, Tessel Bouwens, Esther C. F. Schippers, Toon van Gelderen, Michiel Hilbers, Sander Woutersen, Joost N. H. Reek

**Affiliations:** ^1^ van ‘t Hoff Institute for Molecular Sciences University of Amsterdam Science Park 904 Amsterdam 1098 XH The Netherlands

**Keywords:** artificial photosynthesis, iron, photochemistry, time-resolved spectroscopy, vesicles

## Abstract

Artificial photosynthesis—the direct photochemical generation of hydrogen from water—is a promising but scientifically challenging future technology. Because nature employs membranes for photodriven reactions, the aim of this work is to elucidate the effect of membranes on artificial photocatalysis. To do so, a combination of electrochemistry, photocatalysis, and time‐resolved spectroscopy on vesicle‐embedded [FeFe]hydrogenase mimics, driven by a ruthenium tris‐2,2′‐bipyridine photosensitizer, is reported. The membrane effects encountered can be summarized as follows: the presence of vesicles steers the reactivity of the [FeFe]‐benzodithiolate catalyst towards disproportionation, instead of protonation, due to membrane characteristics, such as providing a constant local effective pH, and concentrating and organizing species inside the membrane. The maximum turnover number is limited by photodegradation of the resting state in the catalytic cycle. Understanding these fundamental productive and destructive pathways in complex photochemical systems allows progress towards the development of efficient artificial leaves.

## Graphical abstract

**Turnover of a new leaf**: A combination of electrochemistry, photocatalysis, and time‐resolved spectroscopy analysis of vesicle‐embedded [FeFe]hydrogenase mimics driven by a ruthenium tris‐2,2′‐bipyridine photosensitizer is reported. Preconcentration in the vesicle membrane leads to different reaction pathways in the photocatalytic proton reduction of the [FeFe]‐benzodithiolate catalyst (see figure).

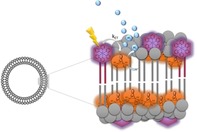

## Introduction

The transition to a sustainable, green energy economy necessitates the development of a corresponding technology through which abundant and renewable resources can be used to generate transportable fuels, such as hydrogen gas. The most straightforward process for the generation of hydrogen is the photolysis of water with sunlight by using bioinspired synthetic photocatalytic systems known as artificial photosynthesis. Since the first working example reported by Fujishima and Honda in 1972,^1^ research into the design and development of these “artificial leaves”^2^ has made spectacular progress,^3^, ^4^, ^5^ up to the point where synthetic and biological systems have started to merge.^6^, ^7^, ^8^, ^9^, ^10^, ^11^, ^12^

Many artificial photosynthetic systems are inspired by the naturally occurring [FeFe]hydrogenase enzyme, which catalyzes proton reduction with high efficiency and turnover numbers (TONs).^13^ Research by the groups of Darensbourg and Lubtiz expanded mechanistic and structural insights into the activity of [FeFe]hydrogenase and showed the importance of the essential protein environment surrounding the [FeFe] core.^14^, ^15^, ^16^, ^17^ Simultaneously, a wide variety of synthetic mimics based on the [FeFe] core of hydrogenase have been developed with different bridgeheads that evolve hydrogen following different mechanisms. To apply hydrogenase mimics in artificial leaves, these catalysts have been studied for their performance in light‐driven hydrogen evolution, but mainly in organic solvents due to the apolar nature of these complexes. Typically, these photocatalytic systems consist of three components: a photosensitizer (PS), a sacrificial electron donor (SED), and the catalyst itself.^18^ The first report on this system by Sun et al. showed that, after reductive quenching of the excited state of [Ru(bpy)_3_]^2+^ (bpy=2,2′‐bipyridine), electron transfer occurred to an azadithiolate‐bridged Fe_2_S_2_ cluster in solution,^19^ leading to a photocatalytic system that yielded up to 5 turnovers,^20^ which was improved upon by Ott et al. through the introduction of a dichlorobenzenedithiolate‐bridged Fe_2_S_2_ cluster, which yielded 200 turnovers.^21^ To avoid the use of organic solvents that might interfere with catalysis, the group of Wu studied this photocatalytic system by using water‐soluble Fe_2_S_2_ analogues, which gave similar TONs and, similar to previous systems, deactivation of the system after 1 to 2 h.^22^, ^23^ On the contrary, the use of intact [FeFe]hydrogenase‐containing cells from *Thiocapsa roseopersicina* in the same [Ru(bpy)_3_]^2+^ system leads to uninterrupted hydrogen evolution for 12 h, with only slow decomposition observed for the isolated enzyme in phosphatidylcholine vesicles.^24^

Evidently, the matrix in which the Fe_2_S_2_ cluster is embedded has an important role in stabilization of the catalyst during photocatalysis, and a synthetic matrix should ideally mimic the function of the original enzyme‐plus‐cell structure. Systems in which hydrogenase mimics were embedded in matrices, such as micelles,^25^, ^26^ amphiphilic polymers,^27^ vesicles,^28^ proteins,^29^ metal–organic frameworks,^30^ and hydrogels,^31^ displayed TONs below 1000, but generally a positive influence of the matrix on the overall efficiency of the catalytic system was reported.

Moreover, photodriven reactions in nature are performed in the presence of a membrane, which consists of amphiphilic molecules that self‐assemble into bilayers in an aqueous environment. The membrane functions can be described as organizing, localizing, and concentrating reactive complexes to enhance reactions and suppress side reactions. In light of this, König and co‐workers assembled both membrane‐embedded water oxidation catalysts and proton reduction catalysts, and showed water oxidation and hydrogen evolution in presence of a [Ru(bpy)_3_]^2+^ as the PS.^28^, ^32^, ^33^

**Figure 1:**
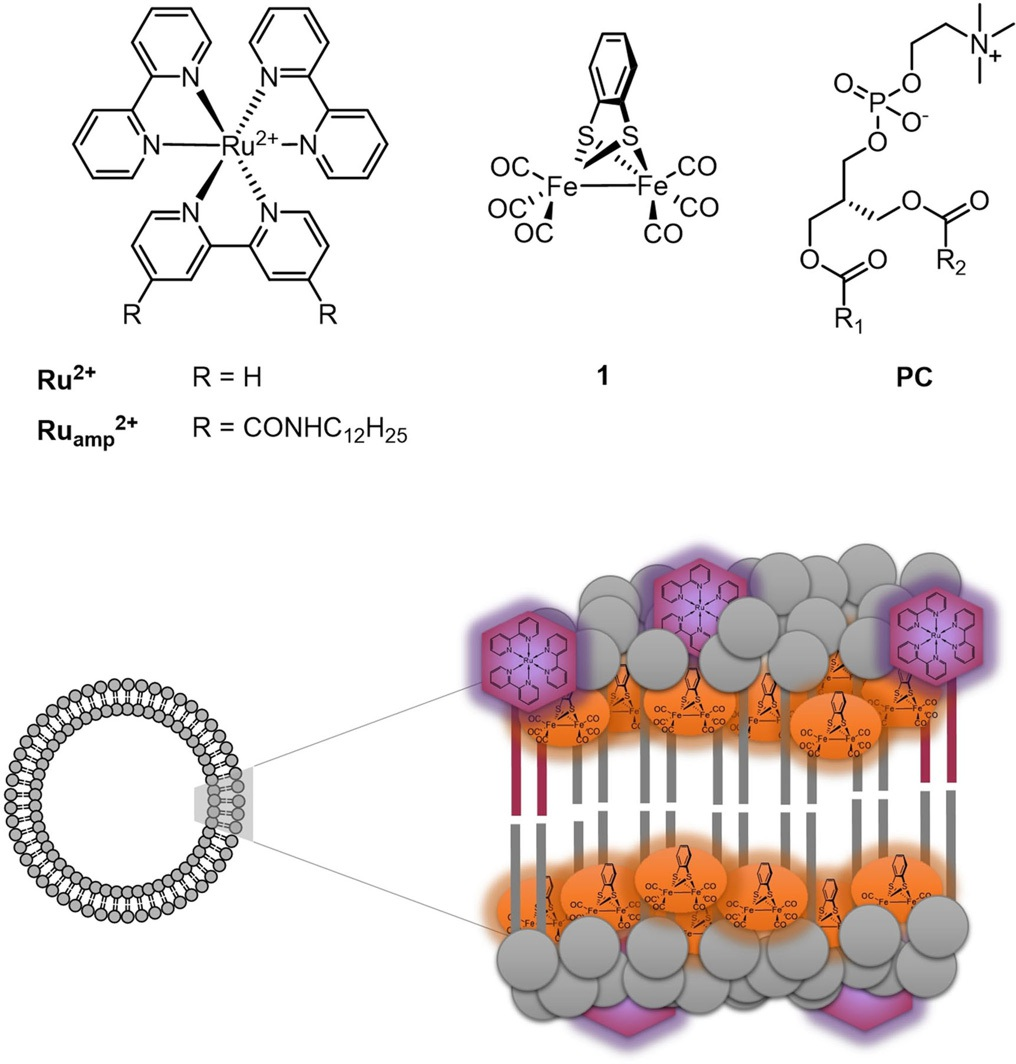
Molecular structures of the components used in the self‐assembled vesicle system for photocatalytic hydrogen production.

Inspired by these results, we were interested in how preorganization of the catalyst and PS components in a lipid bilayer affected the mechanism of (photo)catalytic hydrogen evolution. Herein, we investigated [Ru(bpy)_3_]^2+^ (**Ru^2+^**) as a dye and [(μ‐bdt)Fe_2_(CO)_6_] (**1**; bdt=benzene‐1,2‐dithiolate) as a hydrogen‐evolving catalyst embedded in l‐α‐phosphatidylcholine (**PC**)‐based vesicles (Figure 1), and used a combination of electrochemistry and time‐resolved spectroscopy, visible spectroscopy, and IR spectroscopy to investigate light‐driven proton reduction catalysis.^34^

## Results and Discussion

### Preparation and characterization of the vesicles

We investigated a supramolecular system in which [FeFe]‐benzodithiolate catalyst **1** served as a proton reduction catalyst that was embedded in lecithin‐based vesicles, which were similar in terms of composition and concentrations to the vesicular system reported by König et al.^28^ This system self‐assembles in aqueous buffer solution in either the presence or absence of **1** (details in the Experimental Section). Throughout this research, vesicles are studied under a variety of conditions and the presence of vesicles was confirmed by means of dynamic light scattering (DLS). The vesicles formed were similar over a pH range from 4 to 7, in the presence of **1** (concentrations 0/0.1/0.5 mm), by using a buffer medium (0.1 m ascorbate/phosphate). Small deviations in vesicle diameter are attributed to variations during preparation (Table S1 in the Supporting Information).

**Figure 2:**
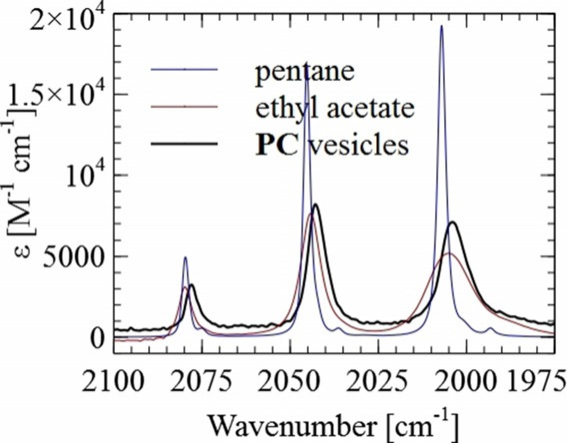
FTIR spectra of 0.5 mm **1** in pentane, ethyl acetate (580 μm path length CaF_2_ cell), and a 0.9 mm solution of **PC** vesicles (25 μm path length CaF_2_ cell) in H_2_O.

Inclusion of **1** in the lipid bilayer was confirmed by using IR spectroscopy (Figure 2). Complex **1** does not dissolve in water and **PC** is used to solubilize this apolar complex in aqueous medium. The iron–carbonyl bands are clearly visible; the three absorption bands of the stretching modes are located at $\tilde{\nu }$ =2078, 2043, and 2004 cm^−1^. Because the width of these bands correlates linearly with the polarity and polarizability solvent parameter π*,^35^ we could use this information to probe the chemical environment of **1** inside the vesicle. The IR spectrum is similar to the spectrum of **1** in ethyl acetate, and quite distinct from that in pentane; this suggests that the carbonyl fragments are in the bilayer in close proximity to the polar head groups of the lipids.

### Electrochemistry

**Figure 3:**
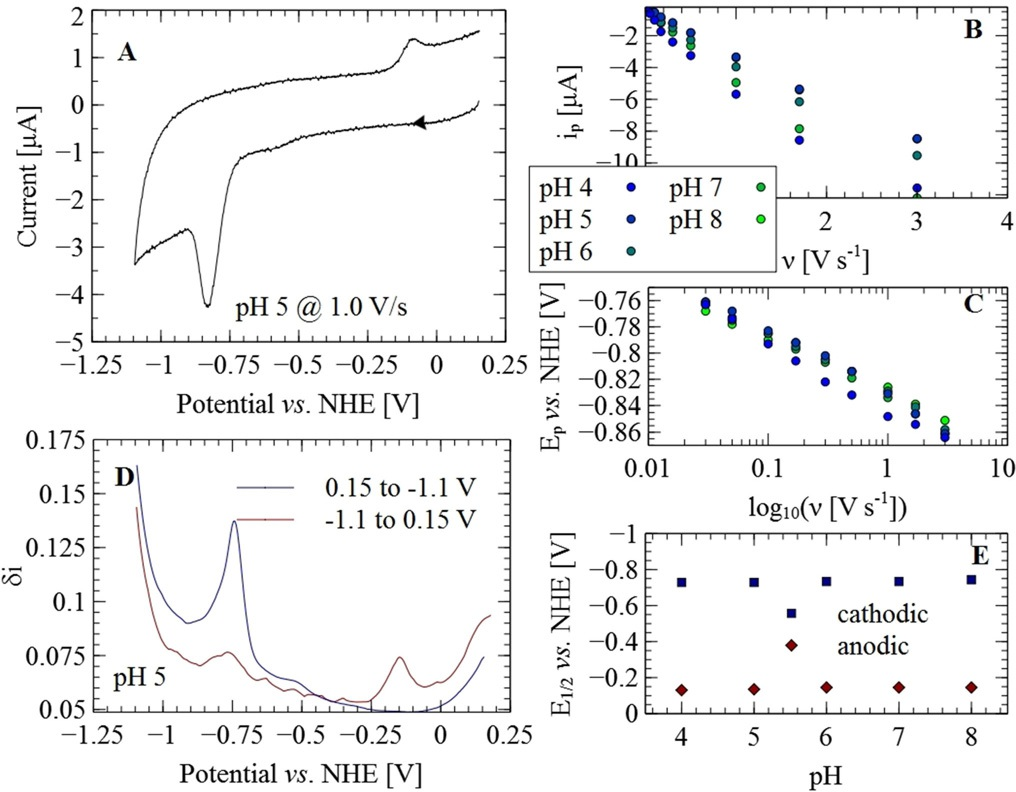
Electrochemical characterization of 0.1 mm **1** in 0.9 mm **PC** vesicles in 0.1 m sodium phosphate buffer at pH values between 4 and 8. A) Cyclic voltammogram at pH 5 and a scan rate of 1.0 V s^−1^. The scan direction is indicated with the arrow. B) Cathodic peak currents versus scan rate at various pH values. C) Cathodic peak potentials versus the logarithm of the scan rate at various pH values. D) Differential pulse voltammetry (DPV) was used to determine the half‐wave potentials of the cathodic and anodic redox processes. E) Cathodic and anodic half‐wave potentials versus pH.

Electrochemical experiments on a 0.1 mm solution of **1** in a solution of vesicles containing 0.1 m sodium phosphate buffer show electrochemical responses similar to that in organic solvents. Complex **1** evolves hydrogen in organic solvents in the presence of acid through double electron transfer followed by protonation (EEC) to form **1**H^−^.^36^ In the presence of strong acid, **1**H^−^ is protonated to evolve H_2_; however, a weak acid is not able to protonate **1**H^−^, and therefore, a second reduction step must take place before protonation can occur. Complex **1** inside vesicles shows a reduction wave with a peak potential at −0.8 V versus a normal hydrogen electrode (NHE; Figure 3 A). Anodic reoxidation at these potentials was not observed; this is indicative of a protonation step after reduction. A reoxidation wave was observed at potentials around −0.1 V versus NHE, which is roughly 0.7 V more positive than that of the reduction event. This behavior is in line with the two‐electron reduction of **1** in organic solvents in the presence of weak acids.^36^, ^37^ Because one step involves protonation, the effect of pH was tested by performing cyclic voltammetry (CV) measurements at pH 4 to 7 (Figures S1–S5 and Tables S2 and S3 in the Supporting Information). The half‐wave potential of the redox processes was determined by means of DPV (Figure 3 D) at −0.73 V versus NHE (which we tentatively assign to a two‐electron reduction process, based on the similarity of the CV results to that of **1** in organic solvent in the presence of a weak acid) and −0.14 V versus NHE (the reoxidation process), irrespective of the pH of the bulk solution (Figure 3 E). The peak potentials and peak currents obtained from CV were also independent of pH. In a previously reported micellar solution of **1**, the reduction process did show a pH dependence on the peak potentials and currents.^38^ This is in contrast with our finding, which indicates that the membrane environment of the vesicles under study provides a constant local effective pH to the catalyst, as observed previously in similar lipid bilayers.^39^

During electrochemical measurements, vesicles appeared to be adsorbed onto the glassy carbon working electrode. This was demonstrated by removing the electrode from the solution after a few scans, rinsing with deionized water, and submerging in fresh buffer solution, after which similar voltammograms were recorded. In the cyclic voltammograms, the cathodic peak currents, *i*_p_, scale linearly with scan rate to the power 0.75 (*ν* ^0.75^; Figure 3 B), which is behavior between that of freely diffusing (*i*_p_ proportional to *ν* ^0.5^) and surface‐adsorbed (*i*_p_ proportional to *ν*) redox‐active species.^40^ This partial diffusion behavior can be attributed to electron hopping through the surface‐adsorbed lipid bilayer mediated by **1** because there is no significant uncompensated for solution resistance during the measurements.^41^, ^42^, ^43^ Overall, the electrochemistry of **1** in vesicles is similar to that of **1** in organic solvent, with respect to redox potentials and protonation behavior.^36^

### Photocatalytic hydrogen production

**Figure 4:**
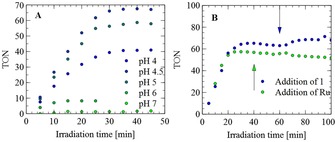
Photocatalytic experiments: irradiation of a solution (5 mL) containing 0.9 mm **PC** vesicles in 0. 1 m ascorbate buffer, 0.1 mm **1**, and 0.2 mm **Ru^2+^**, with *λ*=450 nm LEDs at 4.54 W. A) TON with respect to **1** at different pH values. B) Addition of **1** (at 60 min; pH 4.5) partially restores hydrogen evolution; addition of **Ru^2+^** (at 40 min; pH 5) does not. The TON is expressed with respect to the initial amount of **1**.

Photocatalytic hydrogen evolution from **1** in vesicles was studied by using **Ru^2+^** (in our case [Ru(bpy)_3_]Cl_2_) as the PS and ascorbic acid as the SED. In these experiments, a solution (5 mL) containing 0.1 mm **1** in 0.9 mm **PC** vesicles and 0.2 mm **Ru^2+^** in 0.1 m ascorbate buffer was irradiated with *λ*=450 nm light‐emitting diodes (LEDs) and hydrogen formation was quantified with an in‐line GC setup. Hydrogen evolution started immediately after switching on the LED and ceased within 30 min (Figure 4 A). Control experiments, in which one of the components (**Ru^2+^**, **1**, **PC** or ascorbate) was omitted, did not yield any detectable amounts of hydrogen and demonstrated the necessity of every component in the mixture.

As discussed in the Electrochemistry section, the vesicles provide a constant pH to the catalyst; hence photocatalytic hydrogen evolution in bulk solution can be optimized by fine‐tuning the conditions for the (photoinduced) electron‐transfer steps independently of the hydrogen‐producing part of the system. The pH of the ascorbate buffer was varied between 4 and 7 and an optimum TON of 67 was obtained at pH 4.5. We hypothesize that this pH optimum arises from an increased amount of ascorbate (versus ascorbic acid) at high pH and increased proton reduction activity by **1** at low pH.^44^ We need a high ascorbate concentration because this acts as an electron donor, whereas the protonated species, ascorbic acid, does not. The pH optimum is therefore found to be close to the p*K*_a_ of ascorbic acid, 4.2.^45^ The maximum TON was in all cases achieved within 30 min and was limited by catalyst decomposition, as indicated by IR spectra recorded after the reaction, which no longer showed characteristic iron–carbonyl bands. Also, the addition of 0.5 μmol **1** to the solution after 60 min resulted in extra hydrogen formation (Figure 4 B).

### Time‐resolved UV/Vis and luminescence spectroscopy

**Scheme 1:**
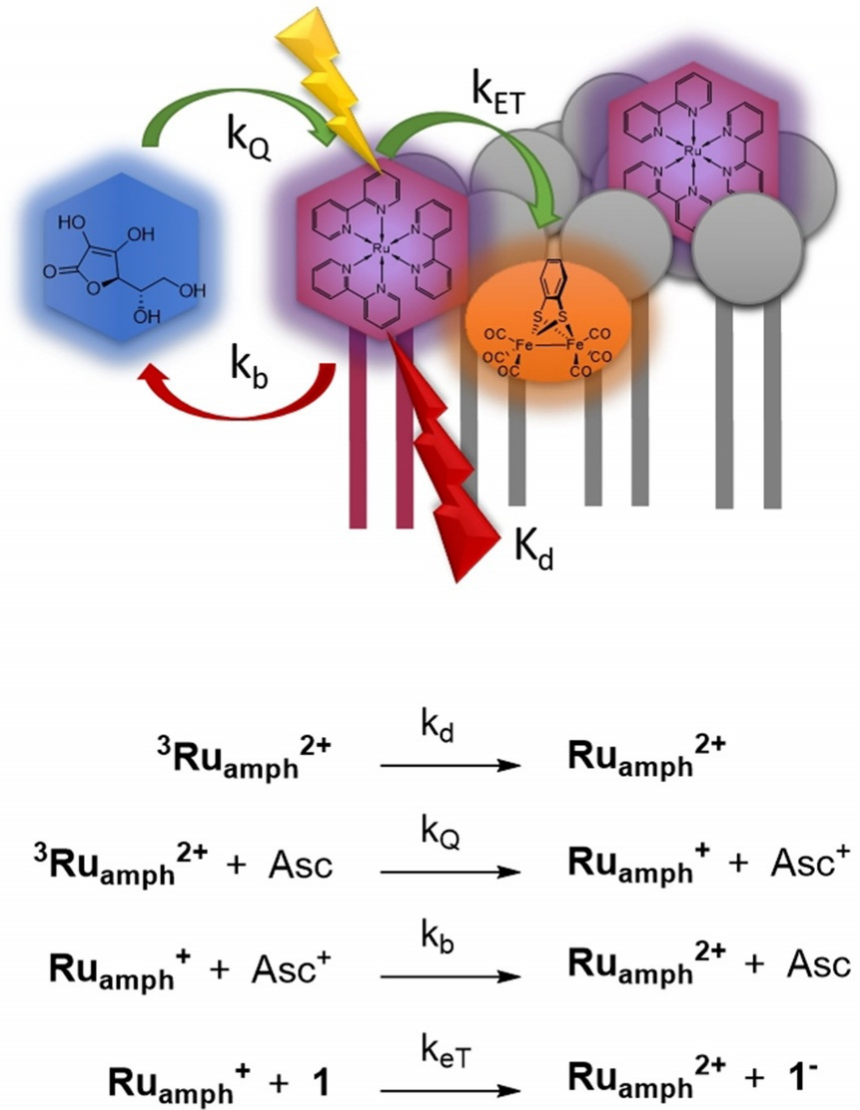
Simplified reactions occurring after the excitation of **Ru_amph_** ^**2+**^ in the presence of ascorbate and **1** in **PC** vesicles.

To confirm our findings from electrochemical studies on weak acid catalysis and to gain an insight in the elementary steps of catalysis, we studied the system by using time‐resolved UV/Vis and luminescence spectroscopy. During these experiments, we aimed to observe the key intermediates shown in Scheme 1: the [^3^Ru(bpy)_3_]^2+^ species after excitation, the [Ru(bpy)_3_]^+^ species after quenching by ascorbate, and reduced **1** after the reaction with [Ru(bpy)_3_]^+^. Excitation of 50 μm **Ru^2+^** in D_2_O with a *λ*=450 nm, 2 mJ, nanosecond pump pulse gave rise to the characteristic triplet species [^3^Ru(bpy)_3_]^2+^, which decayed with a time constant of 677 ns. The same experiment in the presence of the SED ascorbate (0.1 m, pD 4.5) showed the characteristic [Ru(bpy)_3_]^+^ species (*λ*=510 nm) and yielded a quenching rate of *k*_Q_=1.03×10^7^ m ^−1^ s^−1^, which was identical to previously reported rates.^46^ Conducting the same measurement in the presence of **1** in vesicles showed no appreciable difference in the spectra or in the associated decay curves, whereas a faster [Ru(bpy)_3_]^+^ decay was expected if electron transfer from [Ru(bpy)_3_]^+^ to **1** were to occur in appreciable quantities.

To detect reduced **1** after excitation of the **Ru^2+^** PS, time‐resolved IR spectroscopy experiments were performed at the same excitation wavelength (450 nm). The characteristic spectra and kinetics of the PS could again be observed, this time in the CN stretching region between $\tilde{\nu }$ =1400 and 1500 cm^−1^, and matched with data reported in the literature.^47^ No change in the CO stretching region at $\tilde{\nu }$ ≈2000 cm^−1^ was detected for the sample containing **1** in vesicles. This indicates that no significant electron transfer occurs in the time window of these experiments (4 μs), most probably owing to slow diffusion of the photochemically generated reductant, [Ru(bpy)_3_]^+^, to the vesicles.

**Figure 5:**
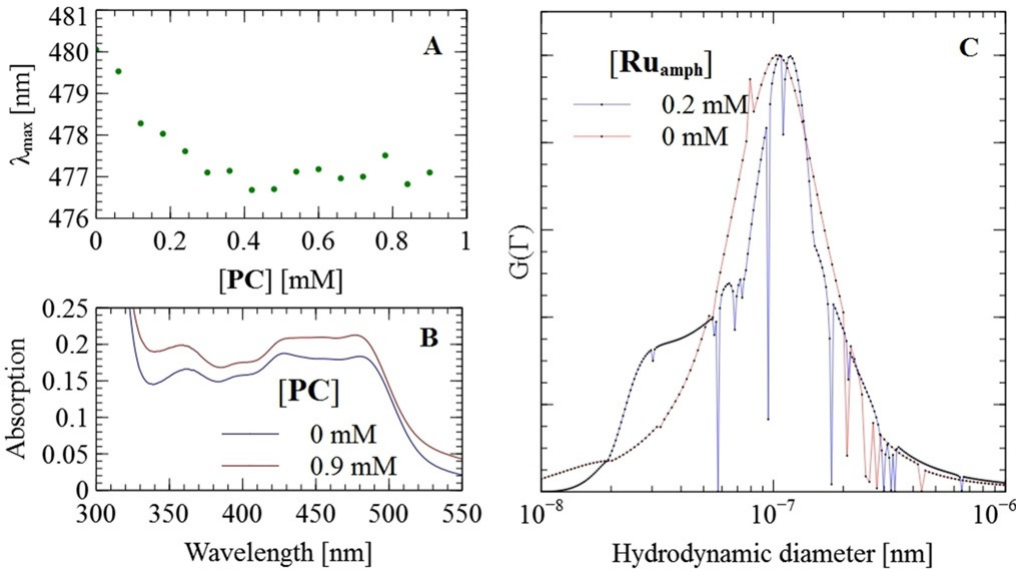
Self‐assembly of the amphiphilic PS **Ru_amph_** ^**2+**^ with **PC** vesicles. A) *λ*_max_ versus **PC** concentration observed by monitoring the metal–ligand charge‐transfer (MLCT) transition at *λ*≈480 nm. B) UV/Vis spectra of **Ru_amph_** ^**2+**^ in the absence and presence of **PC** vesicles. C) Intensity‐weighted distribution of decay rates, as determined by multi‐angle DLS for **PC** vesicles in the absence and presence of **Ru_amph_** ^**2+**^. Details are given in the Supporting Information (ESI 2).

To circumvent this diffusion limitation, and with the aim of probing the reduced forms of **1** after excitation of the sample, we synthesized an amphiphilic analogue of the PS **Ru_amph_** ^**2+**^ (as [Ru(bpy)_2_{5,5′‐(CONHC_12_H_25_)_2_bpy}]Cl_2_; Figure 1) that could be incorporated into vesicles by supramolecular self‐assembly, as shown by König et al.^28^ Because the UV/Vis absorption spectrum of this **Ru^2+^** complex shifts upon changing the polarity of its chemical environment,^48^ we could track the uptake of **Ru_amph_** ^**2+**^ by the vesicles (up to 0.9 mm **PC** to 0.1 mm **Ru_amph_** ^**2+**^). The *D*_H_ at maximum intensity of the **PC** vesicles was 103 nm, which increased to 112 nm upon addition of the PS. This increase cannot be purely attributed to a vesicle size increase, but must be accompanied by a change in the hydrodynamicity of the vesicles upon incorporation of **Ru_amph_** ^**2+**^. The combined UV/Vis and DLS results confirm that the amphiphilic ruthenium PS, **Ru_amph_** ^**2+**^, self‐assembles with the **PC** vesicles upon simple mixing (Figure 5).

The photophysical properties of the ascorbate–**Ru_amph_** ^**2+**^–vesicle–**1** system were studied by means of time‐resolved luminescence and UV/Vis experiments, both in H_2_O and D_2_O at pH/pD 4.35, containing 0.1 mm **Ru_amph_** ^**2+**^. Upon excitation of **Ru_amph_** ^**2+**^ with a 1.0 mJ, *λ*=485 nm, nanosecond pulse, the decay of ^3^ **Ru_amph_** ^**2+**^ and the formation of **Ru_amph_** ^**+**^ are followed over time (Table 1).

**Table 1:** A summary of decay rate constants, *k*_d_, of ^**3**^ **Ru_amph_** ^**2+**^ and ^**3**^ **Ru^2+^** in neat H_2_O and D_2_O, as well as reductive quenching rate constants, *k*_Q_, of ^**3**^ **Ru_amph_** ^**2+**^ (pH/pD 4.35) and ^**3**^ **Ru^2+^** (pD 4.5) by ascorbate in 0.1 m ascorbate buffer.

[**PC**] [mm]	[**1**] [μm]	H_2_O	D_2_O
0	0	*k*_d_=2.68×10^6^ s	*k*_d_=1.54×10^6^ s
		*k*_Q_=1.23×10^8^ m s^−1^	*k*_Q_=1.93×10^8^ m s^−1^
0.9	0	*k*_d_=2.05×10^6^ s	*k*_d_=1.46×10^6^ s
		*k*_Q_=1.08×10^8^ m s^−1^	*k*_Q_=0.61×10^8^ m s^−1^
0.9	50	*k*_Q_=0.84×10^8^ m s^−1^	*k*_Q_=0.57×10^8^ m s^−1^
**Ru^2+^**		*k*_d_=1.64×10^6^ s^49^	*k*_d_=1.05×10^6^ s^49^
		*k*_Q_=0.10×10^8^ m s^−1[46]^	*k*_Q_=0.10×10^8^ m s^−1^

**Figure 6:**
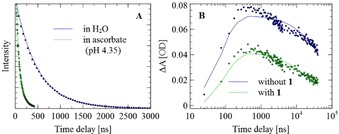
A) Time‐resolved luminescence decay traces (and monoexponential fits) at *λ*=700 nm for samples containing 0.9 mm **PC** vesicles in H_2_O, with and without 0.1 m ascorbate buffer (pH 4.35). B) Time‐resolved UV/Vis traces (and fits to a physical model; see the Supporting Information for more details) at *λ*=515 nm for samples containing 0.9 mm vesicles in 0.1 m ascorbate buffer (H_2_O; pH 4.35) with and without 50 μm **1**.

The triplet lifetime, *τ*_T_, of ^**3**^ **Ru_amph_** ^**2+**^ was determined by fitting the luminescence decay with a monoexponential function (Figure 6 and Figures S6–S9 and S12–S15 in the Supporting Information). The phosphorescence decay rates, *k*_d_=1/*τ*_T_, of ^**3**^ **Ru_amph_** ^**2+**^ were measured in neat (heavy) water and are summarized in Table 1. It can be seen that the decay is faster in H_2_O than that in D_2_O, as is the case for the parent complex **Ru^2+^**.^49^ The presence of vesicles slightly increases the triplet lifetime, as observed previously for similar ruthenium complexes.^50^ The reductive quenching rate, *k*_Q_=(1/*τ*_T_−*k*_d_)/[ascorbate], was calculated from the triplet lifetime in the presence of ascorbate by using the phosphorescence decay rates determined in the absence of quencher. The data summarized in Table 1 show the effect of the hydrogen isotope and the presence of **PC** vesicles on the quenching rate.

The presence of **1** does not seem to affect the quenching rate of ^**3**^ **Ru_amph_** ^**2+**^, which indicates that oxidative quenching of ^**3**^ **Ru_amph_** ^**2+**^ by **1** does not occur to a significant extent. This is in line with the driving force for various pathways and prior studies on the ruthenium–ascorbate couple.^51^

After reductive quenching, the one‐electron reduced form of **Ru_amph_** ^**2+**^_,_ **Ru_amph_** ^+^, is present in solution, which can result in charge recombination with the oxidized form, Asc^+^, of ascorbate or reduce precatalyst **1**.^46^ This first process is back‐electron transfer with bimolecular rate constant *k*_b_. Because these primary electron‐transfer events happen after the reductive quenching of the **Ru_amph_** ^**2+**^ triplet state; this chemistry can be followed by probing the decay of **Ru_amph_** ^+^ through time‐resolved UV/Vis spectroscopy. Because [**Ru_amph_** ^+^]≈[Asc^+^], the value for *k*_b_ of all samples in which **Ru_amph_** ^**+**^ is generated can be estimated with a numerical fit (see Figures S10, S11, S16, and S17 in the Supporting Information).

Table 2 shows the estimation of *k*_b_. For samples in which **1** is absent, the decay directly translates to the back‐electron transfer *k*_b_ rate constant. For samples in which **1** is present, the decay is a result of *k*_b_ and the electron‐transfer rate to **1**, and indeed the decay is faster in the presence of **1**. This observation, in combination with the fact that *k*_Q_ does not change in the presence of **1** (Table 1), indicates that the increased decay is due to an additional decay pathway for **Ru_amph_** ^+^ through electron transfer to **1**. Although phosphorescence decay and the quenching rate constants differ quite substantially between H_2_O and D_2_O, the measured *k*_b_ values do not. Moreover, the difference between *k*_b_ in the presence and absence of **1** is almost identical for H_2_O and D_2_O; therefore, the decay pathways of **Ru_amph_** ^+^ most probably do not involve the transfer of protons. To determine the rate constant, *k*_ET_, for electron transfer from **Ru_amph_** ^+^ to **1**, we assume that the difference in the measured decay values in the absence and presence of **1** is directly related to the electron‐transfer rate via [Asc^+^] (2.13×10^10^−*k*_b_)=[**1**]*k*_ET_. With [Asc^+^] being approximately 1 μm during the monoanion decay, the electron‐transfer rate [**1**]*k*_ET_ is in the order of 10^4^ s^−1^. The concentration of [**1**] in the **PC** vesicles is approximately 70 mm (a concentration increase of over 1000 times; see the Supporting Information for more details), and a lower limit to the bimolecular rate constant, *k*_ET_, in the order of 10^5^ m s^−1^ was determined.

**Table 2:** Back‐electron‐transfer rate constants, *k*_b_, of **Ru_amph_** ^**+**^ with Asc^+^ and **1** in 0.1 m ascorbate buffer (pH/pD 4.35) in the absence and presence of **PC** vesicles and **1**, as determined by means of time‐resolved UV/Vis spectroscopy, and a comparison with the parent compound **Ru^2+^**.

[**PC**] [mm]	[**1**] [μm]	H_2_O	D_2_O
0	0	*k*_b_=2.39×10^10^ m s^−1^	*k*_b_=2.64×10^10^ m s^−1^
0.9	0	*k*_b_=0.73×10^10^ m s^−1^	*k*_b_=0.84×10^10^ m s^−1^
0.9	50	*k*_b_+*k*_ET_ ([**1**]/[Asc^+^])=2.13×10^10^ m s^−1^	*k*_b_+*k*_ET_ ([**1**]/[Asc^+^])=2.28×10^10^ m s^−1^
**Ru^2+^**		*k*_b_=1.8×10^10^ m s^−1[46]^	(not reported)

### Time‐resolved IR spectroscopy and the overall photocatalytic cycle

**Figure 7:**
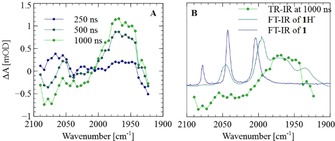
Time‐resolved IR spectroscopy of a sample containing 0.1 mm **1** in 0.9 mm **PC** vesicles and 0.1 mm **Ru_amph_** ^**2+**^ in 0.1 m ascorbate buffer (pD 4.5) in D_2_O in a 250 μm CaF_2_ cell by using a 25 μJ, *λ*=475 nm, nanosecond excitation pulse. A) IR spectra at selected time delays after excitation, showing bleaching of **1** at $\tilde{\nu }$ =2078 cm^−1^ and growth of **1**H^−^ between $\tilde{\nu }$ =1930 and 2000 cm^−1^. B) Time‐resolved spectrum at 1000 ns and comparison with the FTIR spectra of **1** in **PC** vesicles and **1**H^−^ in CH_2_Cl_2_ at 193 K (spectrum from ref. 52).

**Scheme 2:**
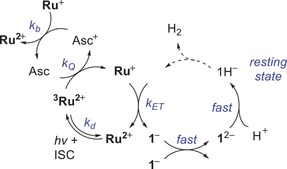
Proposed photocatalytic cycle for hydrogen formation. ISC=intersystem crossing.

To probe the formation of **1^−^** and/or species formed from **1** ^−^ through follow‐up reactivity, time‐resolved IR spectroscopy was performed on a sample containing 0.1 mm **1** in **PC** vesicles and 0.1 mm **Ru_amph_** ^**2+**^ in ascorbate buffer in D_2_O (Figure 7). To avoid catalyst decomposition from the excitation light, the sample was pumped through the cell with a syringe pump. In contrast to experiments in which **Ru^2+^** was used, in this case, we did see a depletion of the lowest energy CO vibration band of **1** at $\tilde{\nu }$ =2078 cm^−1^ and growth of a broad band between $\tilde{\nu }$ =1930 and 2000 cm^−1^ (Figure 7 A). Because depletion of the bands at $\tilde{\nu }$ =2043 and 2004 cm^−1^ belonging to **1** was not observed, the new species formed most likely also contained these bands in its spectrum. Comparing the as‐formed species ($\tilde{\nu }$ =2043, 2000–1930 cm^−1^) with reported reduced and protonated species of **1** gave a unique match with the direduced, monoprotonated species **1**H^−^, which has a reported spectrum of $\tilde{\nu }$ =2045, 1996, 1979, 1963, and 1935 cm^−1^ (Figure 7 B).^52^ This **1**H^−^ species, [(μ,κ^2^‐bdt)(μ‐H)(μ‐CO)Fe_2_(CO)_5_]^−^, is known for being a stable intermediate during weak acid proton reduction catalysis with **1**.^36^ At high initial concentrations of **1**, the formation of this bridging hydride species is thought to occur through a sequence of monoreduction, disproportionation of two monoanions to give **1** and **1** ^2−^, and subsequent protonation of **1** ^2−^ to form **1**H^−^. Because the time‐resolved IR data at delay times shorter than 250 ns are masked by shock waves, we cannot elucidate the chemistry before the formation of **1**H^−^. However, the time‐resolved IR results are in line with the observed electrochemical formation of **1**H^−^ in **PC** vesicles, and thus, we propose the photocatalytic cycle depicted in Scheme 2.

The catalytic steps observed herein are different from those observed by Lomoth and co‐workers in a different Fe_2_S_2_ system in organic solvent (acetonitrile), with which **1** is one‐electron reduced by photogenerated [Ru(dmb)_3_]^+^ (dmb=4,4′‐dimethyl‐2,2′‐bipyridine) to the monoanion **1** ^−^. In contrast to our findings, this species [Fe_2_(bdt)(CO)_6_]^−^ does not disproportionate and persists on the timescale of seconds to react with a strong acid to form **1**H instead.^34^

## Conclusion

A self‐assembled system in which a ruthenium PS and a diiron‐based proton reduction catalyst are preorganized in vesicles was studied in detail to elucidate preorganization effects in the photocatalytic formation of hydrogen by [FeFe]hydrogenase mimics. Electrochemical experiments indicate that the behavior of **1** inside vesicles is similar to that of **1** in organic solvents in the presence of weak acids. Upon irradiation, the PS (**Ru^2+^**) is excited, reductively quenched by ascorbate, and an electron is transferred to **1** to initiate the hydrogen‐evolution cycle. The effects of the vesicle matrix around **1** during catalysis are twofold: 1) we hypothesize that the increased local concentration of **1** leads to faster disproportionation of **1** ^−^ to **1** ^2−^, and 2) the constant neutral pH provided by the vesicles prevents protonation of **1** ^−^. This means that preorganization of the molecular components in vesicles controls the reaction pathway by which the catalyst system photogenerates hydrogen.

Photochemical hydrogen formation at pH 4.5 gives 67 turnovers, and is limited by photodecomposition of the catalyst. The photostability of the hexacarbonyls (and thereby, TON) can possibly be improved upon by substitution of a carbonyl for a phosphorous ligand,^53^ but at the expense of a more negative reduction potential. Photodecomposition can more easily be circumvented by choosing PSs that operate at wavelengths at which the catalytic (resting) species is transparent. As such, the use of [Ru(bpy)_3_]^2+^ with hydrogenase mimics seems to be inappropriate if high TONs are required.

## Experimental Section

### General

All syntheses were carried out under a nitrogen atmosphere by using standard Schlenk techniques. All purifications involving column chromatography were performed in air with non‐degassed solvents. All commercially available chemicals were used as received. l‐α‐Phosphatidylcholine (**PC**; from egg yolk, type XVI‐E, ≥99 %, lyophilized powder) was obtained from Sigma–Aldrich and stored at −20 °C. Compounds **1** and **Ru_amph_** ^**2+**^ were prepared through procedures reported in the literature.^28^, ^54^, ^55^ Solutions of **PC** vesicles were prepared freshly each day. Ascorbate buffer solution was prepared freshly each week and stored at 5 °C; the correct pH/pD was set by mixing of a 0.1 m solution of sodium ascorbate with a 0.1 m solution of ascorbic acid, in which pD was measured with a conventional pH meter by using pD=pH*+0.4, in which pH* is the observed pH value. For the preparation of vesicles, a Vibracell VCX 500 probe‐tip sonicator was used.

### Preparation of PC vesicles

For a 1 mL solution: To a finger flask was added **PC** (5 mg), ethanol (1 mL), or a stock solution of **1** in ethanol (depending on the sample). All solvent was removed on a rotary evaporator until a film was observed. The film was further dried under vacuum and subsequently hydrated by adding buffer solution (1 mL; phosphate buffer or ascorbate buffer) of the desired pH by using a vortex mixer at room temperature. The suspension was transferred to an Eppendorf tube and sonicated by using a probe‐tip sonicator for 1 to 2 min, at 10 s on/5 s off intervals, until the suspension was clear to the eye.

### Steady‐state spectroscopy

The ^1^H NMR spectra were measured on a Bruker AV400 spectrometer. FTIR measurements were conducted on a Bruker ALPHA FTIR spectrometer. UV/Vis measurements were conducted on a HP Agilent 8453 UV/Vis spectrometer.

### Electrochemistry

Cyclic voltammograms and differential pulse voltammograms were performed by using a Metrohm/Autolab PGSTAT128N instrument. The working electrode was a 2 mm diameter glassy carbon disk and a platinum wire counter electrode. The reference electrode (Ag/AgCl) was calibrated against the ferrocyanide couple to obtain potentials versus NHE (see the Supporting Information). Hardware *iR* compensation was employed for all CV measurements. DPV was performed by using a step potential of 5 mV, a modulation potential of 25 mV, a modulation time of 50 ms, and an interval time of 500 ms. Half‐wave potentials were determined from the peak potentials by addition of 12.5 mV (half the modulation potential).

### Photocatalysis

Photocatalysis was performed on a solution (5 mL) in a custom‐built setup, in which the cell headspace (ca. 200 mL volume) was continuously pumped through the sampling valve (25 μL sampling volume) of a Global Analyzer Solutions CompactGC 3.0 gas chromatograph and sampled every 5 min. Irradiation was performed with eight LEDs (*λ*=450 nm; 4.54 W total power) mounted on air‐cooled heat sinks.

### Dynamic light scattering (DLS)

The DLS setup was based on an ALV DLS 5000 goniometer with a digital correlator and a *λ*=633 nm HeNe laser (35 mW) to minimize fluorescence. A typical DLS run was 120 s long and measurements took place at 20 °C. Scattered photons reaching the two photodetectors were cross‐correlated to give one intensity correlation function per measurement. The single‐angle DLS measurements were made at 90°. The multiangle DLS measurements were conducted at 60/70/80/90/95/100/105/110/115/120° and fitted to a set of weighed exponentials by using a nonlinear least‐squares algorithm.

### Time‐resolved luminescence and UV/Vis spectroscopy

In this transient spectroscopy setup, an Ekspla NT342B Nd:YAG laser was used for the generation of the pump light pulse. The probe light was generated by an Excelitas Technologies FX‐1160 high‐stability short‐arc xenon flash lamp, the pulses of which were timed by using a modified PS302 controller from EG&G. The spectrograph used was a Princeton Instruments SpectraPro‐150 instrument. The reference and signal beam were recorded by using a gated, intensified Princeton Instruments PI‐MAX3 charge‐coupled device (CCD) camera. The timing of the excitation pulse, the flash lamp, and the gate of the camera was achieved with a Stanford Research Systems DSG535 delay generator. The samples were measured in a septum‐capped 1 cm quartz cuvette under continuous agitation by a magnetic stirrer bar. Excitation of 50 μm **Ru^2+^** in D_2_O with a *λ*=450 nm, 2 mJ, nanosecond pump pulse gave rise to the characteristic triplet species, [^3^Ru(bpy)_3_]^2+^ (*λ*=370/565/820 nm), which decayed with a time constant of 677 ns. Excitation of the samples with a 1.0 mJ, *λ*=485 nm, nanosecond pulse gave a ^3^ **Ru_amph_** ^**2+**^ concentration between 1 and 3 μm (depending on the sample) at *t*=0. For all samples, both luminescence and absorption spectra were recorded at time delays up to 4 (luminescence) or 40 μs (absorption). Kinetic analysis was performed by global fitting of the spectral data with a set of equations derived from the system of chemical reactions or by numerically solving of the system of ordinary differential equations inside the target function for the nonlinear least‐squares routine (see the Supporting Information for a full explanation and derivation of formulae). As such, all (e.g., pre‐exponential) constants had physical meaning, and the data were fitted with a physical description of the system (see below). Time‐resolved absorption spectra can be found in Figures S6–S19 in the Supporting Information. The contents of the samples are summarized in Table 3.

**Table 3:** Samples measured in the time‐resolved UV/Vis and luminescence study. All five samples were prepared and measured in both H_2_O and D_2_O.

Sample	[**Ru_amph_** ^**2+**^]/[**PC**]/[**1**]/[Asc] [mm/mm/μm/m]	Luminescence	UV/Vis
1	0.1/0/0/0	*k*_d_/liquid	
2	0.1/0/0/0.1	*k*_Q_/liquid	*k*_b_/liquid
3	0.1/0.9/0/0	*k*_d_/vesicles	
4	0.1/.9/0/0.1	*k*_Q_/vesicles	*k*_b_/vesicles
5	01/0.9/50/0.1		*k*_ET_/vesicles

### Time‐resolved IR spectroscopy

A commercial Spectra‐Physics OPA‐800C BBO‐based optical parametric amplifier (OPA) was pumped by a Spectra‐Physics Hurricane Ti:sapphire laser (*λ*=800 nm; 480 μJ) with a repetition rate of 1 kHz. IR probe pulses were generated by a difference‐frequency mixing signal and idler from the OPA in a AgGaS_2_ crystal. The nanosecond visible pump pulses (*λ*=475 nm; 25 μJ) were generated in a GWU versaScan‐L BBO‐based optical parametric oscillator (OPO) pumped by a Spectra‐Physics Quanta‐Ray INDI Nd:YAG laser with a repetition rate of 20 Hz. The sample cell with CaF_2_ windows spaced by 250 μm was placed in the IR focus and the sample was pumped through the cell at a flow rate of 10 μL min^−1^ by using a syringe pump. A custom‐built 30 pixel HgCdTe (MCT) detector coupled to an Oriel MS260i spectrograph was employed to record the transient spectra by subtracting nonpumped absorption spectra from the pumped absorption spectra. Background correction was performed by subtracting the time‐averaged spectra obtained at negative time delays.

### UV/Vis study on the incorporation of Ru_amph_^2+^ into vesicles

Because the UV/Vis absorption spectrum of this complex was sensitive to the polarity of its chemical environment,^48^ we tracked the UV/Vis spectral changes upon the addition of vesicles (up to 0.9 mm **PC**) to an aqueous solution of 0.1 mm **Ru_amph_** ^**2+**^. (A proper supramolecular titration was not possible because the absolute absorption values were irreproducible due to light scattering from the vesicles.) The peak position of the MLCT transition at *λ*≈480 nm was used as an indicator for the binding of **Ru_amph_** ^**2+**^ to the vesicles (see the Supporting Information, ESI 2).^56^

### Concentration of compounds in/on liposomes

The concentration of **PC** in vesicles was [**PC**]=1000 mL L^−1^/(768 g mol^−1^×0.99 mL g^−1^)=1.3 m.^57^ The concentration of **1** in **PC** vesicles was 50 μm/0.9 mm×1.3 m=70 mm. The concentration of **Ru_amph_** ^**2+**^ in **PC** vesicles was 100 μm/0.9 mm×1.3 m=140 mm.

### Chemical models and kinetic equations

**Samples containing only Ru^2+^**: For the samples only containing the PS, there were only two chemical species present, namely, the ground state (G) and the excited state (E). From *t=*0 onward, the reactivity was E→G with decay constant *k*_d_.

The rate equations and boundary conditions are given in Table 4, and the resulting concentration time dependence is given in Equations (1), (2).

(1) $$E\left(t\right)=C\;\exp\left(-k{}_{d}t\right)$$

(2) $$G\left(t\right)=-C\;\exp\left(-k{}_{d}t\right)$$

**Table 4:** A summary of the rate equations and boundary conditions of samples containing only **Ru^2+^**.

Species	Rate equation	Boundary condition
E	dE(*t*)/d*t*=−*k*_d_E(*t*)	*E*(0)=*C*
G	G(*t*)=−E(*t*)

**Samples containing Ru^2+^ and ascorbate**: With respect to the previous sample, one additional species was now present. This “monoanion” (M) was generated and consumed as given by Equations (3), (4), (5).

(3) $$E\to G,\;\mathrm{with}\;\mathrm{decay}\;\mathrm{constant}\;k{}_{d}$$

(4) $$E+\mathrm{Asc}\to M+\mathrm{Asc}{}^{+},\;\mathrm{with}\;\mathrm{quenching}\;\mathrm{constant}\;k{}_{Q}$$

(5) $$M+\mathrm{Asc}{}^{+}\to G+\mathrm{Asc},\;\mathrm{with}\;\mathrm{constant}\;k{}_{b}$$

Because [Asc]≫[**Ru^2+^**], we could assume that [Asc]=Q was constant over time. Moreover, because Asc^+^ was generated and consumed stoichiometrically with M, we could equate [Asc^+^](*t*)=[M](*t*). The rate equations and boundary conditions are given in Table 5.

**Table 5:** A summary of the rate equations and boundary conditions of samples containing **Ru^2+^** and ascorbate.

Species	Rate equation	Boundary condition
E	dE(*t*)/d*t*=−(*k*_d_+*k*_Q_ *Q*)E(*t*)	*E*(0)=*C*
M	dM(*t*)/d*t*=*k*_Q_ *Q*E(*t*)−*k*_b_M(*t*)^2^	*M* ^0^=0
G	G(*t*)=−E(*t*)−M(*t*)

The resulting concentration time‐dependence could only be expressed analytically for E(*t*) [Eq. (6).

(6) $$E\left(t\right)=C\;\exp[-\left(k{}_{d}+k{}_{Q}Q\right)t]$$

For monoanion decay, *M*(*t*), a Riccati equation [Eq. (7)] resulted, which could not be solved analytically.

(7) $$\mathrm{dM}\left(t\right)/dt=k{}_{Q}QC\;\exp\left[-\left(k{}_{d}+k{}_{Q}Q\right)t\right]-k{}_{b}M\left(t\right){}^{2}$$

However, given values for the constants *k*_Q_ *Q*, *k*_d_ and *k*_b_, and setting *M* ^0^=0, the function M(*t*) could be evaluated numerically, for which we used the Matlab function ode45. After evaluation of M(*t*), the ground‐state function, G(*t*), could be evaluated through G(*t*)=−E(*t*)−M(*t*).

### Fitting of time‐resolved luminescence and UV/Vis data

All acquired time‐resolved data was fitted in Matlab by using the nonlinear least‐squares function lsqcurvefit in the physical model outlined above. The experimental time‐resolved data matrix *A*_exp_(*λ*;*t*) was fitted to *A*_fit_(*λ*;*t*), which was a linear combination of time‐dependent species spectra as given by Equation (8).

(8) $$A{}_{\mathrm{fit}}\left(\lambda ;t\right)=\epsilon {}_{G}\left(\lambda \right)G\left(t\right)+\epsilon {}_{E}\left(\lambda \right)E\left(t\right)+\epsilon {}_{M}\left(\lambda \right)M\left(t\right)$$

The spectra *ϵ*_species_(*λ*) and constants *k*_Q_ *Q*, *k*_d_, and *k*_b_ were determined by the fitting procedure, if they had not already been determined in previous experiments. This yielded an approach in which the set of experiments were designed in such a way that every experiment generated a set of spectra and rate constants that could be used as fixed values in the next experiment, to provide minimum freedom during the fitting procedure, and thereby, maximum accuracy in the determination of kinetic rate constants. To minimize the amount of parameters in the fitting procedure even further, we only determined difference spectra with respect to G because G(*t*)=−E(*t*)−M(*t*) [Eq. (9)].

(9) $$A{}_{\mathrm{fit}}\left(\lambda ;t\right)=\left[\epsilon {}_{E}\left(\lambda \right)-\epsilon {}_{G}\left(\lambda \right)\right]E\left(t\right)+\left[\epsilon {}_{M}\left(\lambda \right)-\epsilon {}_{G}\left(\lambda \right)\right]M\left(t\right)$$

This redundancy of *ϵ*_G_(*λ*) was general and held for all analyzed chemical systems.

The values of the obtained rate constants can be found in Tables 1 and 2. The Supporting Information contains cyclic voltammograms and experimental, fitted, and error plots for all time‐resolved spectroscopy experiments.

## Conflict of interest

The authors declare no conflict of interest.

## Supporting information

As a service to our authors and readers, this journal provides supporting information supplied by the authors. Such materials are peer reviewed and may be re‐organized for online delivery, but are not copy‐edited or typeset. Technical support issues arising from supporting information (other than missing files) should be addressed to the authors.

Supplementary
