# Supplementary material for: Photocatalytic Hydrogen Generation by Vesicle‐Embedded [FeFe]Hydrogenase Mimics: A Mechanistic Study
Source: Chemistry. 2019 Sep 26;25(61):13921–9. doi: 10.1002/chem.201902514 (PMC6899470; doi:10.1002/chem.201902514)
Supplement: Supplementary file 1 — Supplementary [file CHEM-25-13921-s001.pdf]

CHEMISTRY
A European Journal

Supporting Information

Photocatalytic Hydrogen Generation by Vesicle-Embedded [FeFe]Hydrogenase Mimics: A Mechanistic Study

René Becker+, Tessel Bouwens+, Esther C. F. Schippers, Toon van Gelderen, Michiel Hilbers, Sander Woutersen,* and Joost N. H. Reek*[a]

chem_201902514_sm_miscellaneous_information.pdf

Contents

1. Liposomes Size at Different Preparation Conditions 3
2. Clarification on Titration of $\mathbf{R_{amph}}^{2+}$ 3
3. Electrochemistry of **1** inside Liposomes 4
4. Time-resolved luminescence for sample 1 9
5. Time-resolved luminescence for sample 2 10
6. Time-resolved UV-vis for sample 2 11
7. Time-resolved luminescence for sample 3 12
8. Time-resolved luminescence for sample 4 13
9. Time-resolved UV-vis for sample 4 14
10. Time-resolved UV-vis for sample 5 16
11. References 18

1. Liposomes Size at Different Preparation Conditions

Table S1: Liposome size at different conditions. PC = phosphatidylcholine (type XIII-E from egg yolk), buffer concentration is 0.1 M.

**Sample**	**Complex 1 (μM)**	**pH**	**Buffer**	**Lecithin source**	**Radius (nm)**	**P.D.I .**
**A**	0	4.5	Ascorbate	99% PC	36.3	0.31
**B**	100	4.5	Ascorbate	99% PC	57.9	0.39
**C**	83	4.5	Ascorbate	99% PC	39.00	0.39
**D**	100	7	Ascorbate	99% PC	57.2	0.34
**E**	100	7	Ascorbate	60% PC	63.3	0.35
**F**	0	4	Phosphate	60% PC	66.2	0.35
**G**	0	7	Phosphate	60% PC	40.2	0.29
**H**	100	7	Phosphate	60% PC	54.5	0.29
**I**	500	4	Phosphate	60% PC	65.0	0.33

2. Clarification on Titration of $\mathbf{Ru_{amph}^{2+}}$

The peak position of the MLCT transition around 480 nm was used as an indicator for binding of $\mathbf{Ru_{amph}^{2+}}$ to the vesicles. An almost linear shift (from 480 to 477 nm) was observed going from 0 to 0.4 mM **PC**, which then remained constant at 477 nm up to 0.9 mM **PC** (Figure 5A and Figure 5B). To probe further interactions of the ruthenium amphiphile with the vesicles, the hydrodynamic diameter D_H of the vesicles was determined by multi-angle dynamic light scattering (MDLS) on a single vesicle sample, before and after addition of $\mathbf{Ru_{amph}^{2+}}$. Inversion of the MDLS autocorrelation function was performed by non-linear least squares fitting of 250 to 500 weighted exponentials over a diameter range of 10^{-8} to 10^{-4} m with logarithmically spaced intervals.[S1]

3. Electrochemistry of 1 inside Liposomes

Electrochemical studies of 1 inside liposomes was performed at varying scan rates at pH 4–8. The results are presented in Figure S1–S5 in which the y-axes expressed in current divided by scan rate to show all CVs in one figure. In spurely diffusisve systems it is a common method to plot the current devidied by the square root of the scan rate when scan rate dependent study is conducted. Our findings support that we are not dealing with a purely diffusive system, hence this scan rate dependent study is represented as current divided by scan rate.

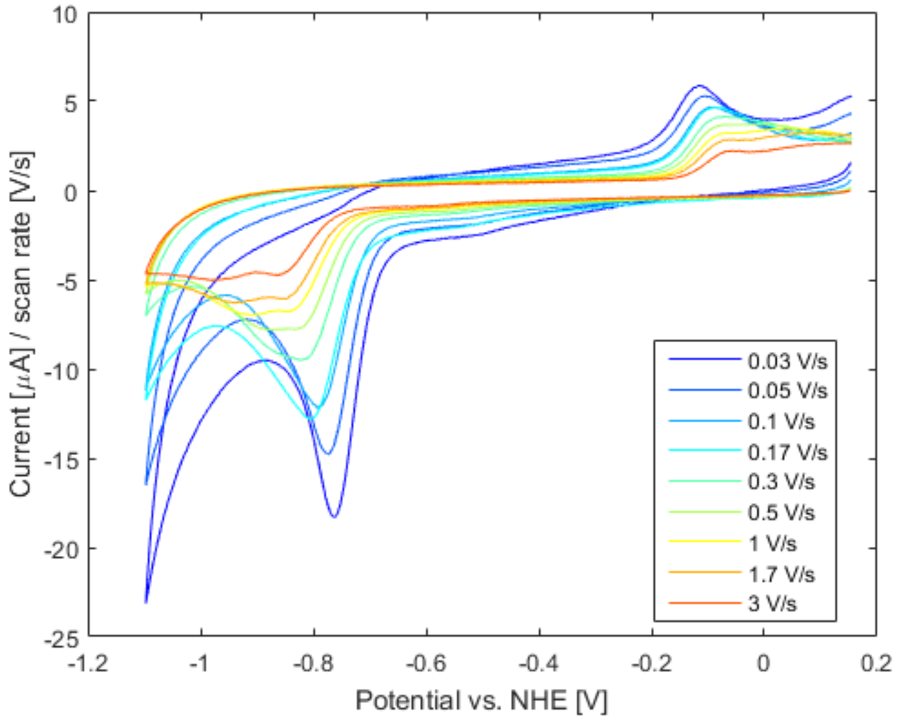

Figure S1: Cyclic voltammograms at pH 4 with on the y-axes current divided by scan rate.

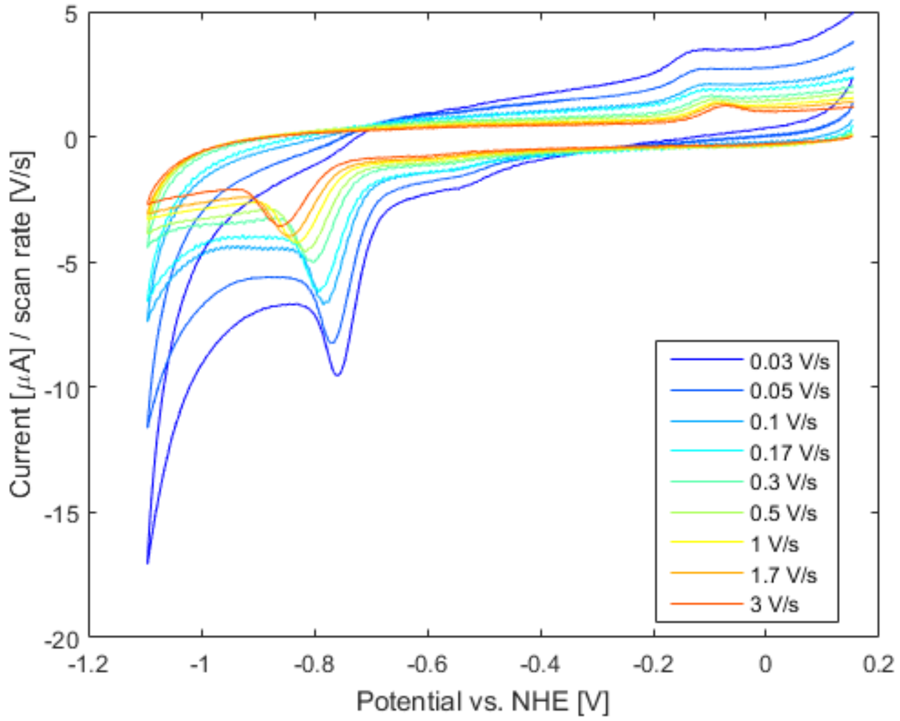

Figure S2: Cyclic voltammograms at pH 5 plotted with on the y-axes current divided by scan rate.

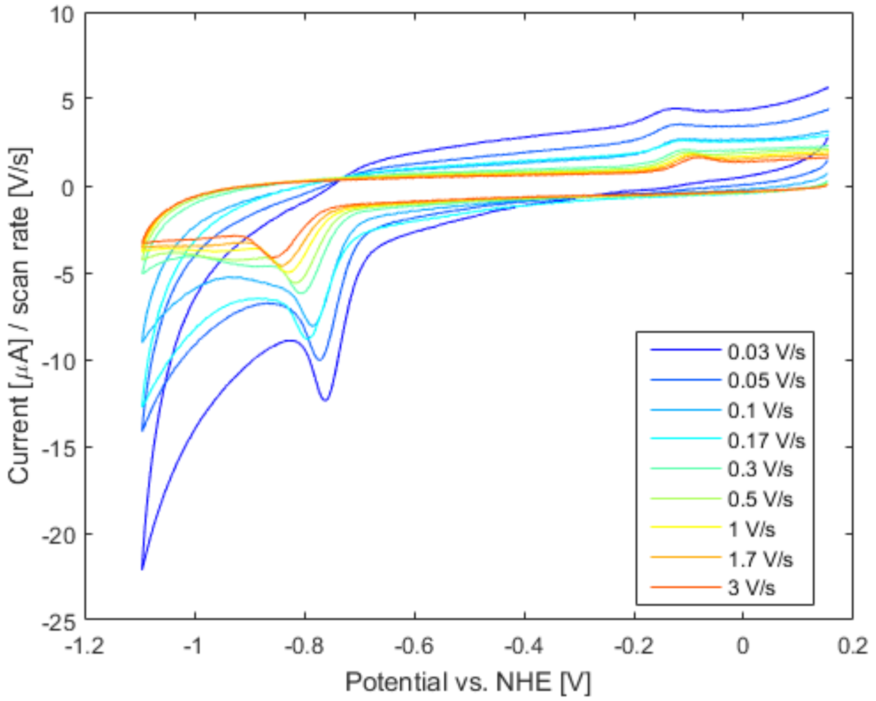

Figure S3: Cyclic voltammograms at pH 6 plotted with on the y-axes current divided by scan rate.

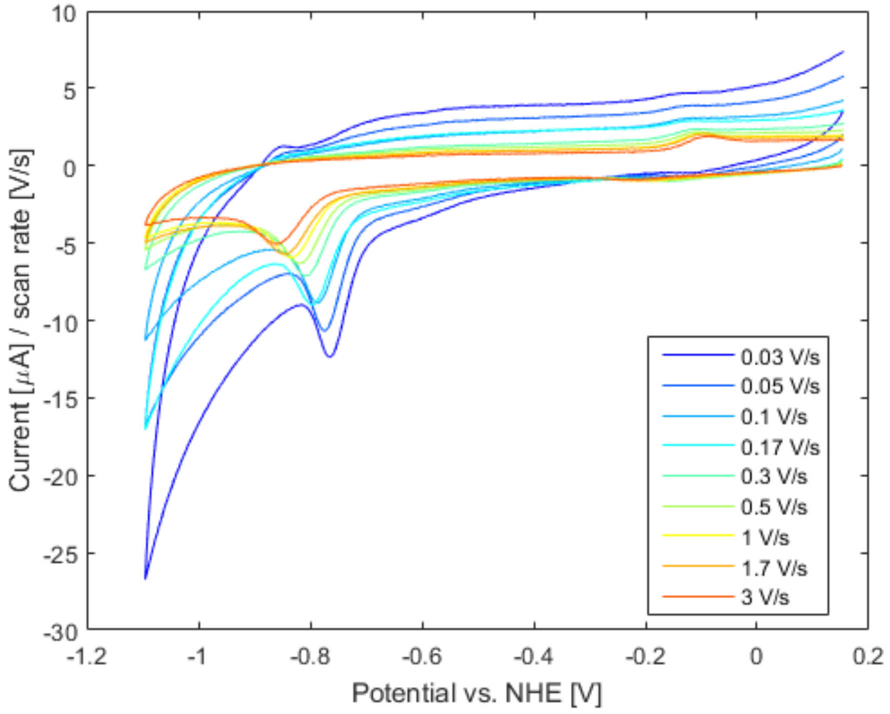

Figure S4: Cyclic voltammograms at pH 7 plotted with on the y-axes current divided by scan rate.

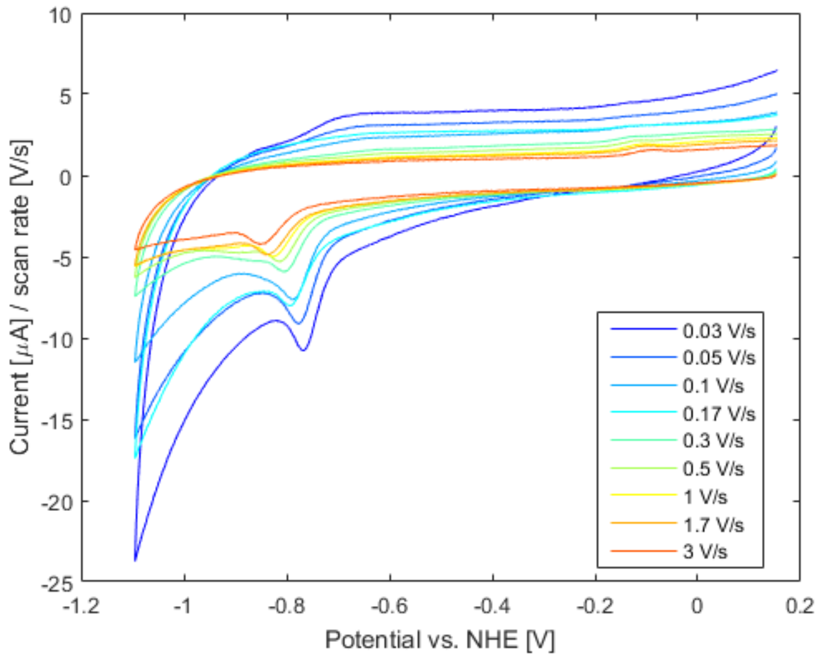

Figure S5: Cyclic voltammograms at pH 8 plotted with on the y-axes current divided by scan rate.

Table S2: Cathodic peak potentials (V versus NHE) versus scan rate and pH.

	pH 4	pH 5	pH 6	pH 7	pH 8
0.03 V/s	-0.763	-0.761	-0.761	-0.763	-0.768
0.05 V/s	-0.774	-0.768	-0.773	-0.775	-0.778
0.1 V/s	-0.793	-0.783	-0.785	-0.785	-0.79
0.17 V/s	-0.806	-0.792	-0.795	-0.797	-0.792
0.3 V/s	-0.822	-0.802	-0.805	-0.807	-0.805
0.5 V/s	-0.832	-0.814	-0.814	-0.819	-0.814
1 V/s	-0.848	-0.831	-0.829	-0.834	-0.826
1.7 V/s	-0.854	-0.846	-0.841	-0.846	-0.839
3 V/s	-0.864	-0.861	-0.858	-0.861	-0.851

Table S3: Cathodic peak currents (μA) versus scan rate and pH.

	pH 4	pH 5	pH 6	pH 7	pH 8
0.03 V/s	-0.44	-0.22	-0.29	-0.28	-0.21
0.05 V/s	-0.61	-0.33	-0.41	-0.43	-0.32
0.1 V/s	-1.02	-0.54	-0.66	-0.73	-0.55
0.17 V/s	-1.75	-0.82	-1.16	-1.20	-0.91
0.3 V/s	-2.40	-1.18	-1.51	-1.78	-1.24
0.5 V/s	-3.25	-1.81	-2.26	-2.64	-1.83
1 V/s	-5.68	-3.34	-3.96	-4.95	-3.38
1.7 V/s	-8.57	-5.36	-6.15	-7.85	-5.41
3 V/s	-11.57	-8.47	-9.53	-12.19	-8.49

Reference potential of the Ag/AgCl reference electrode

To determine the reference potential of the reference electrode in the phosphate buffer solution versus NHE, the standard couple $Fe(CN)_6^{3-}$/ $Fe(CN)_6^{4-}$ is measured. CVs at different pH values at different scan rates were recorded to check the stability of the reference electrode. We found that the reduction potential of $Fe(CN)_6^{3-}$ did not vary with pH and was found at $E_{1/2}$ = 0.205 V. According to literature, the standard potential E^0 of this couple is 0.36 V versus NHE.[S2, S3] Thus, 0.155 V must be added to all potentials obtained to relate the potential versus NHE.

4. Time-resolved luminescence for sample 1

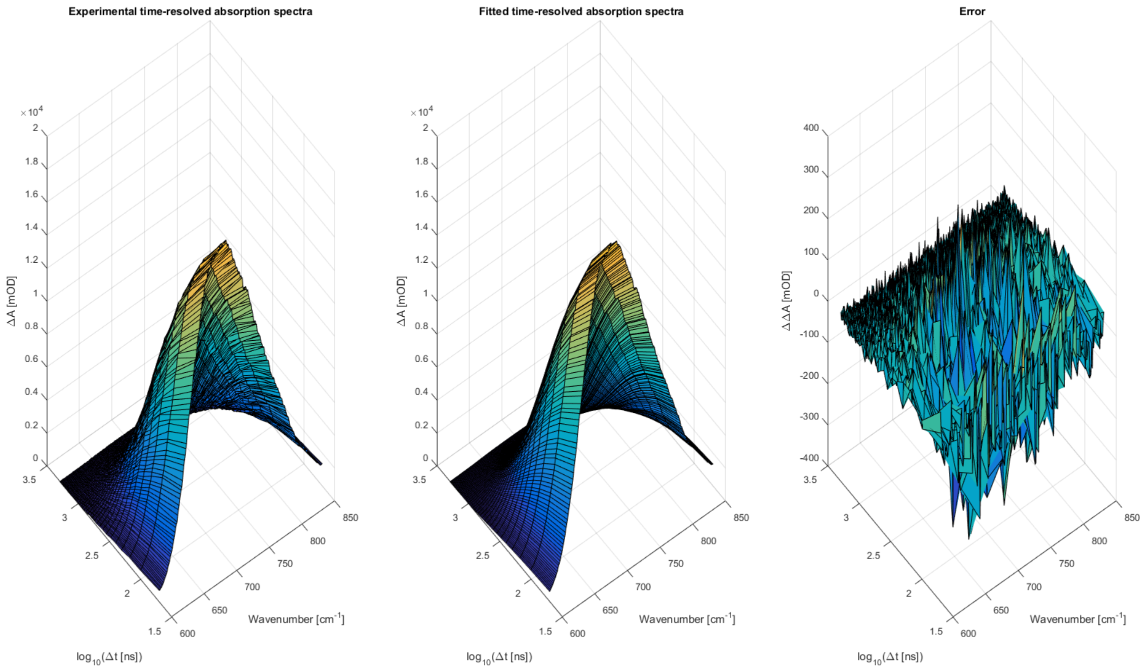

Figure S6: Time-resolved luminescence for sample 1 in H_2O. Sample 1 contains 0.1 mM $\mathbf{Ru_{amph}}^{2+}$ in absence of **PC**, **1** and Asc to observe the decay of $^{3}\mathbf{Ru_{amph}}^{2+}$ to $\mathbf{Ru_{amph}}^{2+}$ (k_d in liquid).

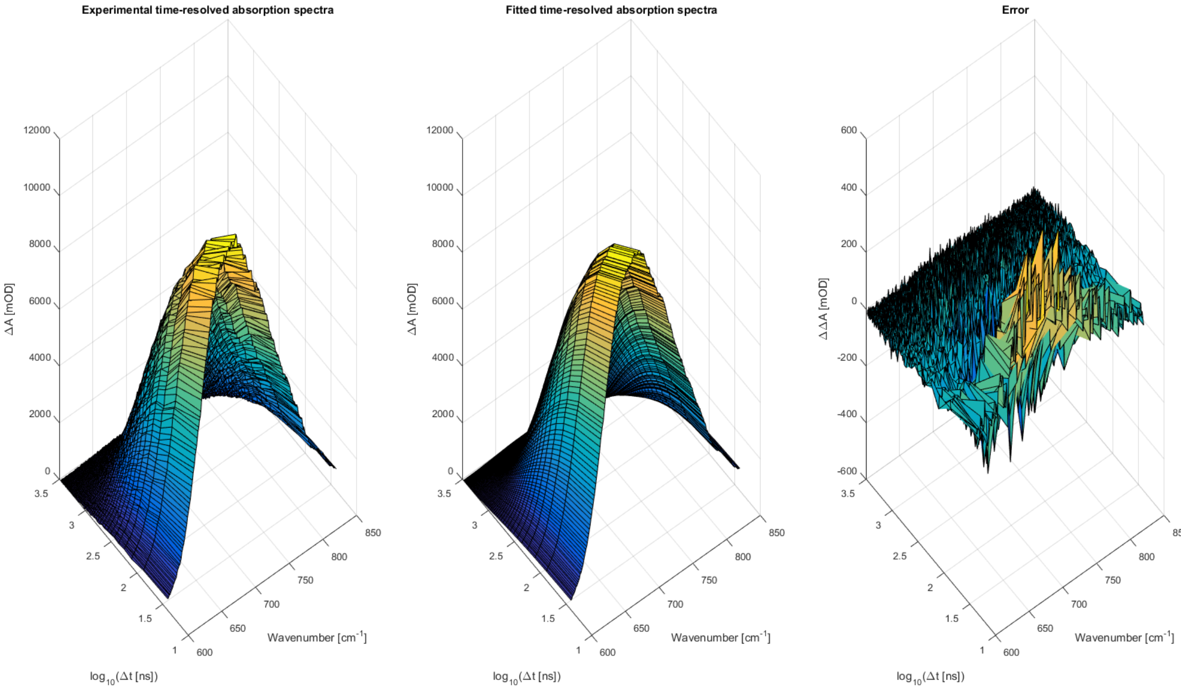

Figure S7: Time-resolved luminescence for sample 1 in D_2O. This sample contains 0.1 mM $\mathbf{Ru_{amph}}^{2+}$ in absence of **PC**, **1** and Asc to observe the decay of $^{3}\mathbf{Ru_{amph}}^{2+}$ to $\mathbf{Ru_{amph}}^{2+}$(k_d in liquid).

5. Time-resolved luminescence for sample 2

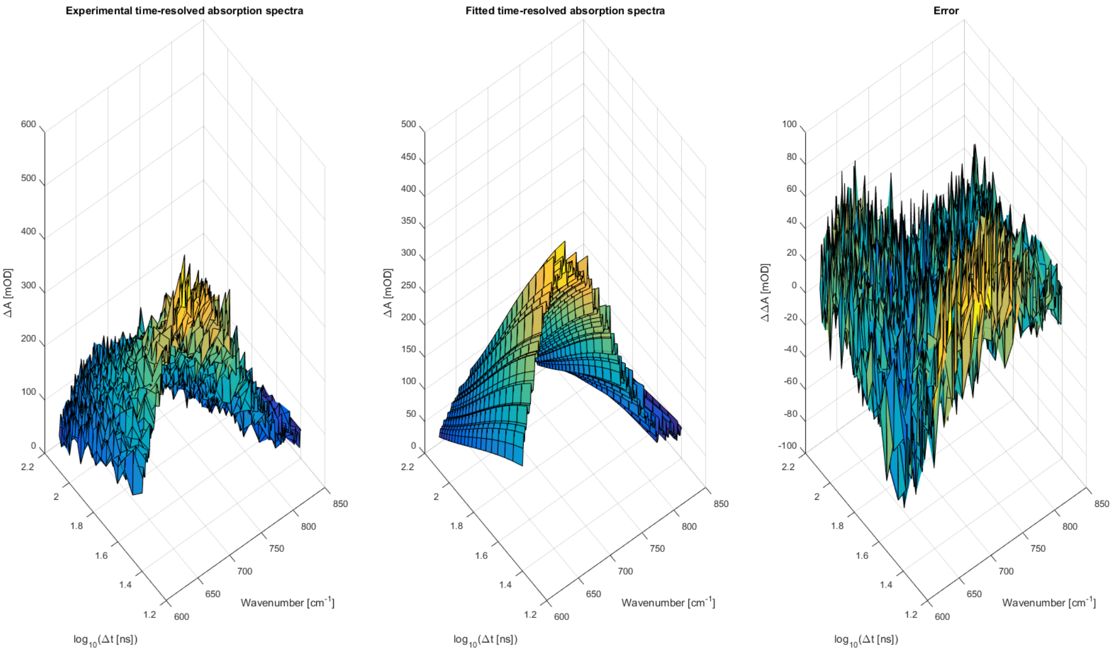

Figure S8: Time-resolved luminescence for sample 2 in H_2O. This sample contains 0.1 mM **Ru**$_{\mathbf{amph}}^{2+}$ and 0.1 M ascorbate in absence of **PC**, and **1** to observe the decay of ^{3}**Ru**$_{\mathbf{amph}}^{2+}$ in presence of ascorbate (k_Q in liquid).

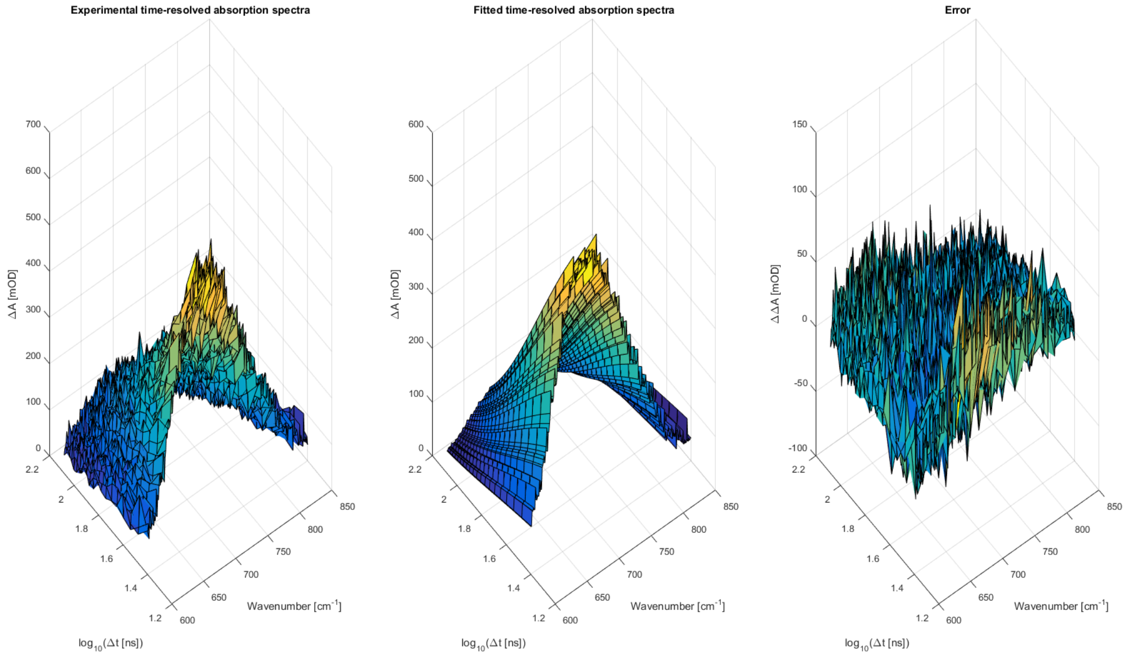

Figure S9: Time-resolved luminescence for sample 2 in D_2O. This sample contains 0.1 mM **Ru**$_{\mathbf{amph}}^{2+}$ and 0.1 M ascorbate in absence of **PC**, and **1** to observe the decay of ^{3}**Ru**$_{\mathbf{amph}}^{2+}$ in presence of ascorbate (k_Q in liquid).

6. Time-resolved UV-vis for sample 2

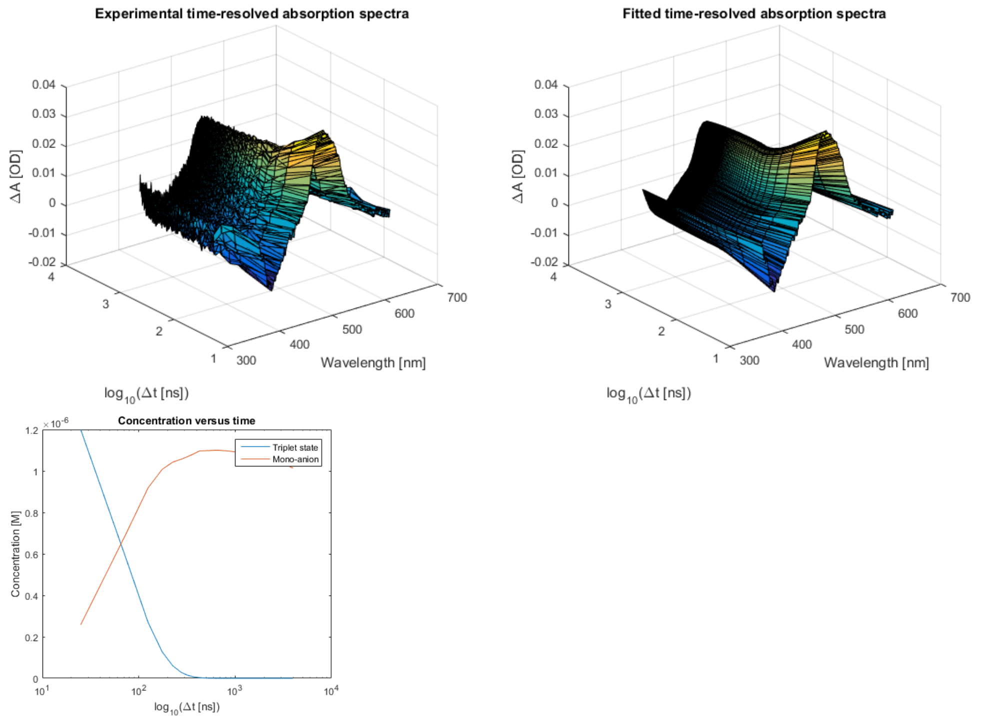

Figure S10: Time-resolved UV-vis for sample 2 in H_2O. This sample contains 0.1 mM **Ru**$_{\mathbf{amph}}^{\mathbf{2+}}$ and 0.1 M ascorbate in absence of **PC**, and **1** to observe the decay of **Ru**$_{\mathbf{amph}}^{\mathbf{+}}$ in presence of ascorbate (k_b in liquid).

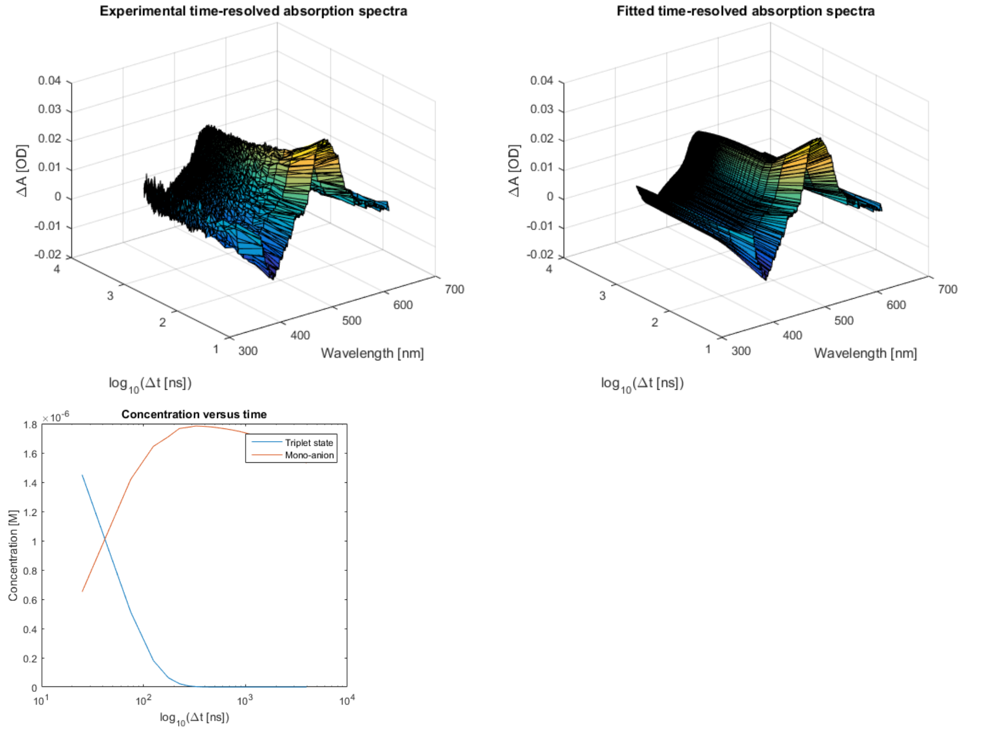

Figure S11: Time-resolved UV-vis for sample 2 in D_2O. This sample contains 0.1 mM **Ru**$_{\mathbf{amph}}^{\mathbf{2+}}$ and 0.1 M ascorbate in absence of **PC**, and **1** to observe the quenching decay of **Ru**$_{\mathbf{amph}}^{\mathbf{+}}$ in presence of ascorbate (k_b in liquid).

7. Time-resolved luminescence for sample 3

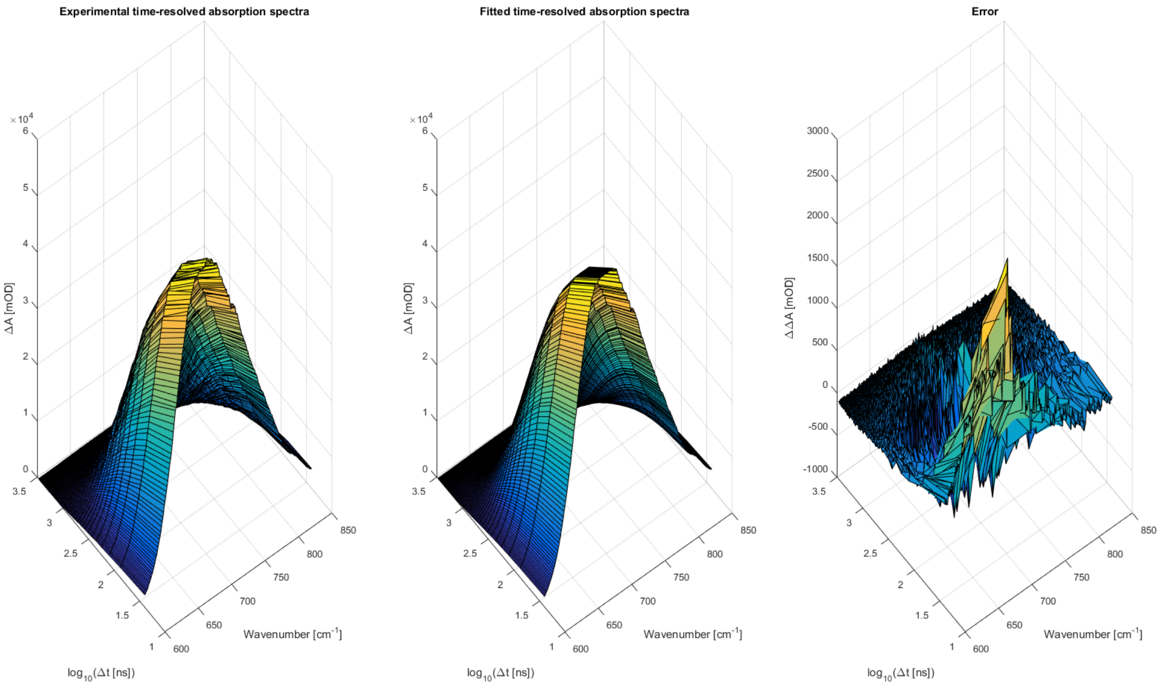

Figure S12: Time-resolved luminescence for sample 3 in H_2O. This sample contains 0.1 mM $\mathbf{Ru_{amph}}^{2+}$ and 0.9 mM **PC** in absence of **1** and Asc to observe the decay of $^3\mathbf{Ru_{amph}}^{2+}$ to $\mathbf{Ru_{amph}}^{2+}$ in vesicles. (k_d).

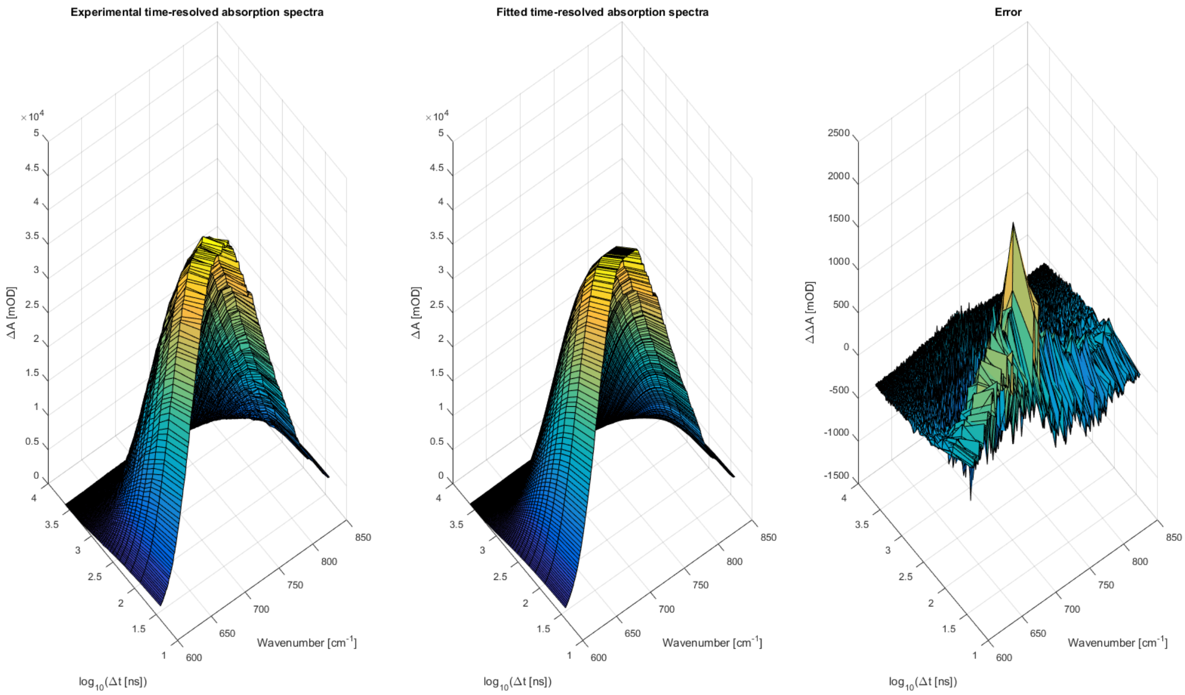

Figure S13: Time-resolved luminescence for sample 3 in D_2O. This sample contains 0.1 mM $\mathbf{Ru_{amph}}^{2+}$ and 0.9 mM **PC** in absence of **1** and Asc to observe the decay of $^3\mathbf{Ru_{amph}}^{2+}$ to $\mathbf{Ru_{amph}}^{2+}$ in vesicles (k_d).

8. Time-resolved luminescence for sample 4

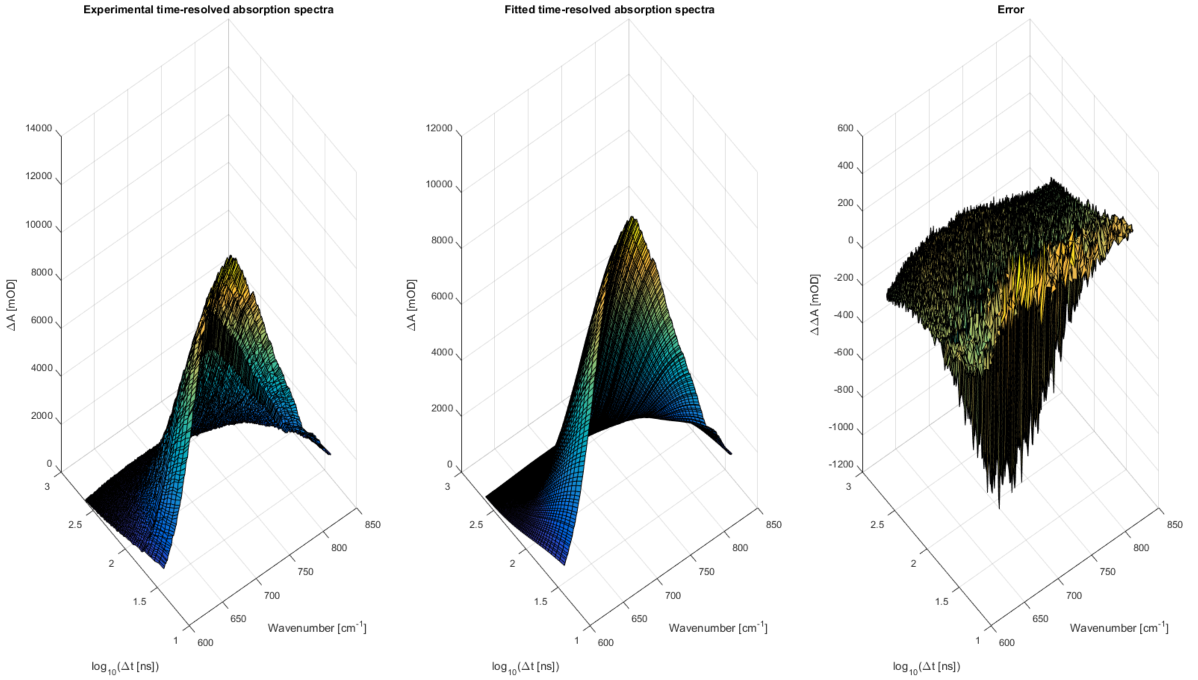

Figure S14: Time-resolved luminescence for sample 4 in H_2O. This sample contains 0.1 mM $\mathbf{Ru_{amph}}^{2+}$, 0.9 mM **PC** 0.1 M ascorbate in absence of **1** to observe the decay of $^3\mathbf{Ru_{amph}}^{2+}$ in presence of ascorbate in vesicles (k_Q and k_b).

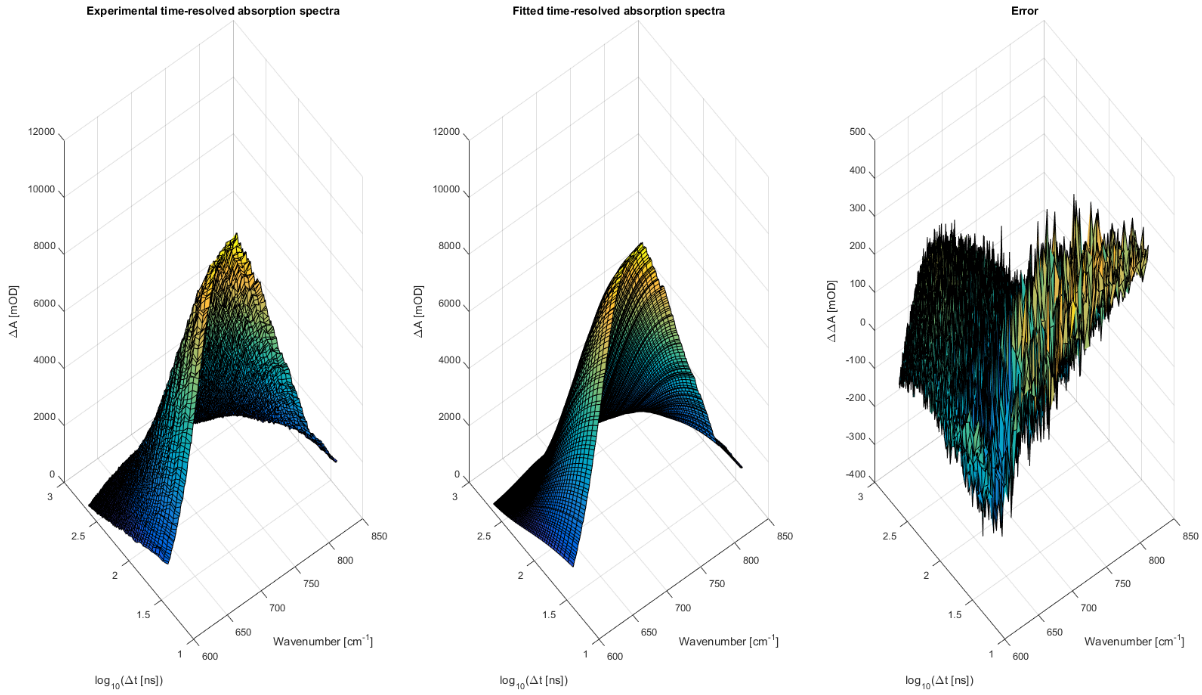

Figure S15: Time-resolved luminescence for sample 4 in D_2O. This sample contains 0.1 mM $\mathbf{Ru_{amph}}^{2+}$, 0.9 mM **PC** 0.1 M ascorbate in absence of **1** to observe the decay of $^3\mathbf{Ru_{amph}}^{2+}$ in presence of ascorbate in vesicles (k_Q and k_b).

9. Time-resolved UV-vis for sample 4

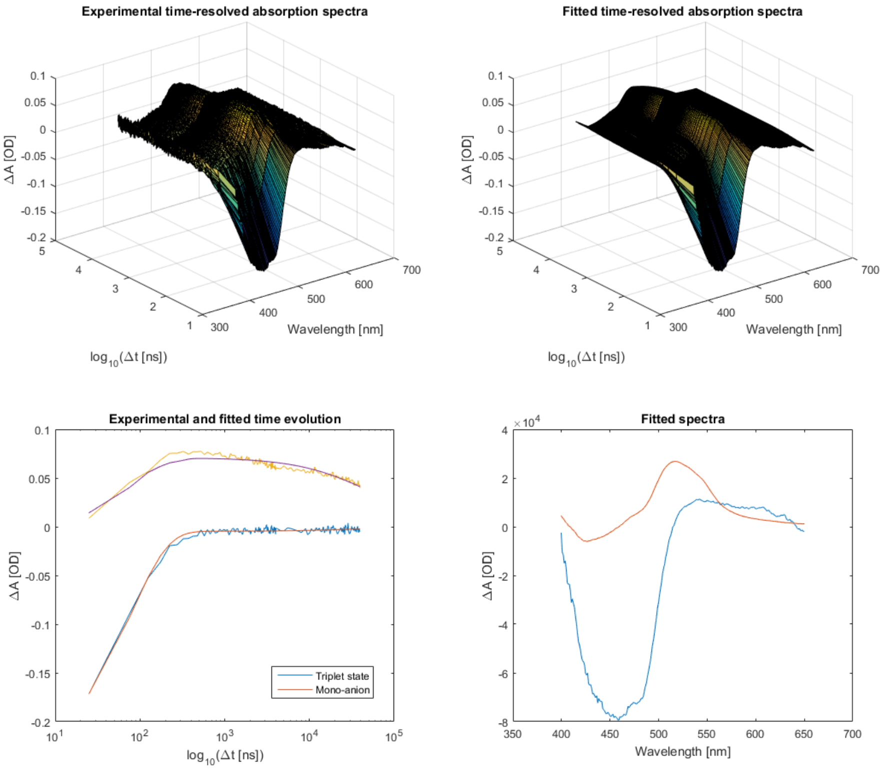

Figure S16: Time-resolved UV-vis for sample 4 in H_2O. This sample contains 0.1 mM **Ru**$_{\mathbf{amph}}^{\mathbf{2+}}$**,** 0.9 mM **PC** 0.1 M ascorbate in absence of **1** to observe the decay of **Ru**$_{\mathbf{amph}}^{+}$ in presence of ascorbate in vesicles (k_b).

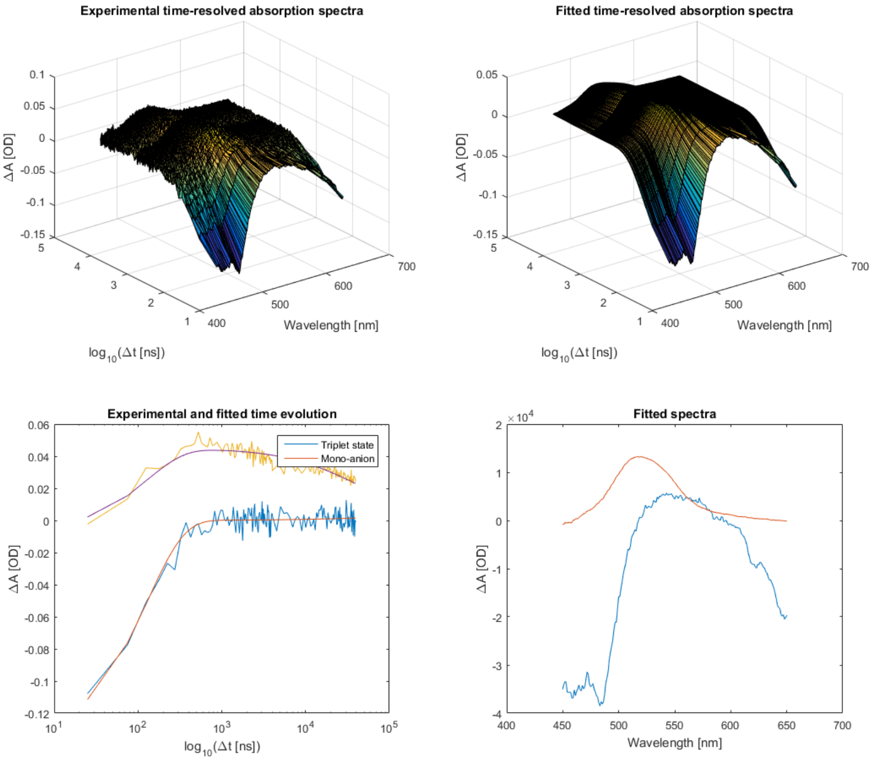

Figure S17: Time-resolved UV-vis for sample 4 in D_2O. This sample contains 0.1 mM $\mathbf{Ru_{amph}}^{2+}$**,** 0.9 mM **PC** 0.1 M ascorbate in absence of **1** to observe the decay of $^{3}\mathbf{Ru_{amph}}^{2+}$ in presence of ascorbate in vesicles (k_b).

10. Time-resolved UV-vis for sample 5

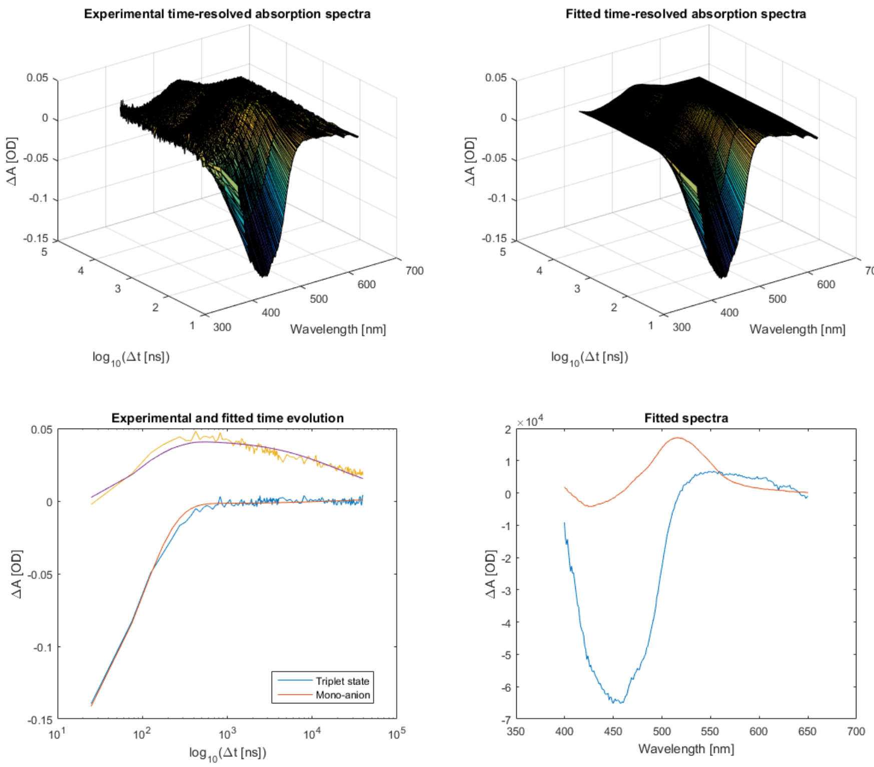

Figure S18: Time-resolved UV-vis for sample 5 in H_2O. This sample contains 0.1 mM $\mathbf{Ru_{amph}}^{2+}$, 0.9 mM **PC,** 0.1 M ascorbate and 50 µM **1** to observe the decay of $\mathbf{Ru_{amph}}^{+}$ in presence of ascorbate in vesicles (k_{ET}).

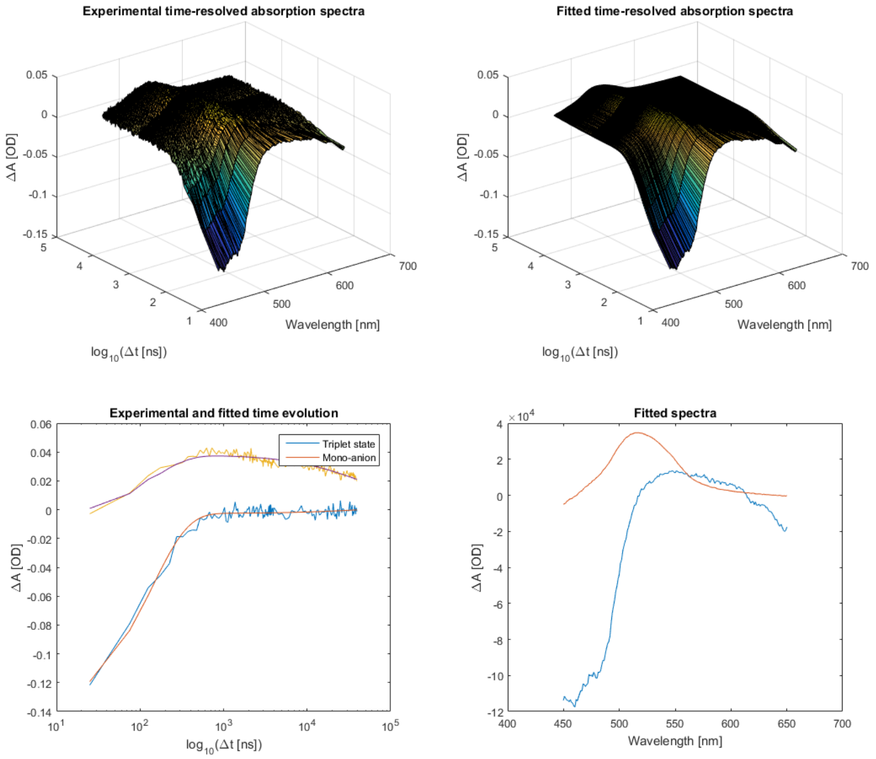

Figure S19: Time-resolved UV-vis for sample 5 in D_2O. This sample contains 0.1 mM **Ru$_{amph}$$^{2+}$**, 0.9 mM **PC,** 0.1 M ascorbate and 50 µM **1** to observe the decay of **Ru$_{amph}$$^{+}$** in presence of ascorbate in vesicles (k_{ET}) .

11. References

[S1] M. Naiim, A. Boualem, C. Ferre, M. Jabloun, A. Jalocha, P. Ravier, *Soft Matter* **2015**, *11*, 28–32.

[S2] P. A. Rock *J. Phys. Chem.* **1966**, *70*, 576–580.

[S3] A. J. Bard, L. R. Faulkner *Electrochemical Methods*; John Wiley & Sons, Inc.: Hoboken, 2001, p. 808.
